# MIIND : A Model-Agnostic Simulator of Neural Populations

**DOI:** 10.3389/fninf.2021.614881

**Published:** 2021-07-06

**Authors:** Hugh Osborne, Yi Ming Lai, Mikkel Elle Lepperød, David Sichau, Lukas Deutz, Marc de Kamps

**Affiliations:** ^1^Institute for Artificial Intelligence and Biological Computation, School of Computing, University of Leeds, Leeds, United Kingdom; ^2^School of Medicine, University of Nottingham, Nottingham, United Kingdom; ^3^Centre for Integrative Neuroplasticity, University of Oslo, Oslo, Norway; ^4^Department of Computer Science, Eidgenössische Technische Hochschule Zurich, Zurich, Switzerland; ^5^School of Computing and Leeds Institute for Data Analytics, University of Leeds, Leeds, United Kingdom

**Keywords:** simulator, neural population, population density, software, Python, dynamical systems, network, GPU

## Abstract

MIIND is a software platform for easily and efficiently simulating the behaviour of interacting populations of point neurons governed by any 1D or 2D dynamical system. The simulator is entirely agnostic to the underlying neuron model of each population and provides an intuitive method for controlling the amount of noise which can significantly affect the overall behaviour. A network of populations can be set up quickly and easily using MIIND's XML-style simulation file format describing simulation parameters such as how populations interact, transmission delays, post-synaptic potentials, and what output to record. During simulation, a visual display of each population's state is provided for immediate feedback of the behaviour and population activity can be output to a file or passed to a Python script for further processing. The Python support also means that MIIND can be integrated into other software such as The Virtual Brain. MIIND's population density technique is a geometric and visual method for describing the activity of each neuron population which encourages a deep consideration of the dynamics of the neuron model and provides insight into how the behaviour of each population is affected by the behaviour of its neighbours in the network. For 1D neuron models, MIIND performs far better than direct simulation solutions for large populations. For 2D models, performance comparison is more nuanced but the population density approach still confers certain advantages over direct simulation. MIIND can be used to build neural systems that bridge the scales between an individual neuron model and a population network. This allows researchers to maintain a plausible path back from mesoscopic to microscopic scales while minimising the complexity of managing large numbers of interconnected neurons. In this paper, we introduce the MIIND system, its usage, and provide implementation details where appropriate.

## 1. Introduction

### 1.1. Population-Level Modeling

Structures in the brain at various scales can be approximated by simple neural population networks based on commonly observed neural connections. There are a great number of techniques to simulate the behaviour of neural populations with varying degrees of granularity and computational efficiency. At the highest detail, individual neurons can be modelled with multiple compartments, transport mechanisms, and other biophysical attributes. Simulators such as GENESIS (Wilson et al., 1988; Bower and Beeman, 2012) and NEURON (Hines and Carnevale, 2001) have been used for investigations of the cerebellar microcircuit (D'Angelo et al., 2016) and a thalamocortical network model (Traub et al., 2005). Techniques which simulate the individual behaviour of point neurons such as in NEST (Gewaltig and Diesmann, 2007), or the neuromorphic system SpiNNaker (Furber et al., 2014), allow neurons to be individually parameterised and connections to be heterogeneous. This is particularly useful for analysing information transfer such as edge detection in the visual cortex. They can also be used to analyse so called finite-size effects where population behaviour only occurs as a result of a specific realisation of individual neuron behaviour. There are, however, performance limitations on very large populations in terms of both computation speed and memory requirements for storing the spike history of each neuron.

At a less granular level, rate-based techniques are a widely used practice of modeling neural activity with a single variable, whose evolution is often described by first-order ordinary differential equations, which goes back to Wilson and Cowan (1972). The Virtual Brain (TVB) uses these types of models to represent activity of large regions (nodes) in whole brain networks to generate efficient simulations (Sanz Leon et al., 2013; Jirsa et al., 2014). TVB demonstrates the benefits of a rate based approach with the Epileptor neural population model yielding impressive clinical results (Proix et al., 2017). The Epileptor model is based on the well-known Hindmarsh-Rose neuron model (Hindmarsh and Rose, 1984). However, the behaviour of this and other rate based models is defined at the population level instead of behaviour emerging from a definition of the underlying neurons. Therefore, these models have less power to explain simulated behaviours at the microscopic level.

Between these two extremes of granularity is a research area which bridges the scales by deriving population level behaviour from the behaviour of the underlying neurons. So called population density techniques (PDTs) have been used for many years (Knight, 1972; Knight et al., 1996; Omurtag et al., 2000) to describe a population of neurons in terms of a probability density function. The transfer function of a neuron model or even an experimental neural recording can be used to approximate the response from a population using this technique (Wilson and Cowan, 1972; El Boustani and Destexhe, 2009; Carlu et al., 2020). However, analytical solutions are often limited to regular spiking behaviour with constant or slowly changing input. The software we present here, MIIND, provides a numerical solution for populations of neurons with potentially complex behaviours (for example bursting) receiving rapidly changing noisy input with arbitrary jump sizes. The noise is usually assumed to be shot noise, but MIIND can also be used with other renewal processes such as Gamma distributed input (Lai and de Kamps, 2017). It contains a number of features that make it particularly suitable for dynamical systems representing neuronal dynamics, such as an adequate handling of boundary conditions that emerge from the presence of thresholds and reset mechanisms, but is not restricted to neural systems. The dynamical systems can be grouped in large networks, which can be seen as the model of a neural circuit at the population level.

**Figure 1:**
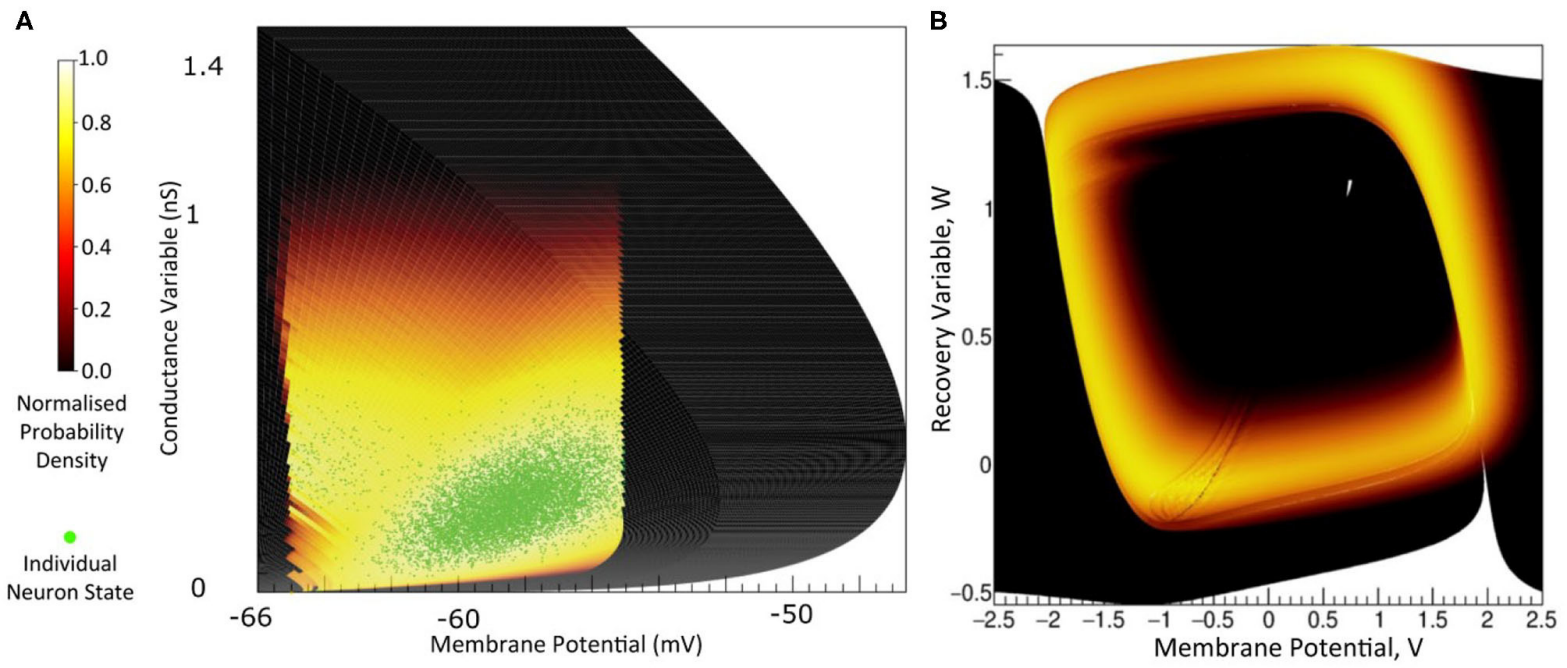
**(A)** The state space of a conductance based point model neuron. It is spanned by two variables: the membrane potential and a variable representing how open the channel is. This channel has an equilibrium potential that is positive. The green dots represent the state of individual neurons in a population. They are the result of the direct simulation of a group of neurons. MIIND, however, produces the heat plot representing a density (normalised to the maximum density value) which predicts where neurons in the population are likely to be: most likely in the white areas, least likely in the red areas and not at all in the black areas. The sharp vertical cut of the coloured area at −55 mV represents the threshold at which neurons are removed from state space. They are subsequently inserted at the reset potential, at their original conductance state value. **(B)** The state space of a Fitzhugh-Nagumo neuron model. The axes have arbitrary units for variables *V* and *W*. There is no threshold-reset mechanism and the density follows a limit cycle. After a certain amount of simulation time, neurons can be found at all points along the limit cycle as shown here by a consistently high brightness.

The key idea behind MIIND is shown in Figure 1A. Here, a population of neurons is simulated. In this case, the neurons are defined by a conductance based leaky-integrate-and-fire neuron model with membrane potential and state of the conductance as the two variables. The neuron's evolution through state space is given by a two-dimensional dynamical system described by Equation (1).

(1) $$\begin{array}{l} \begin{array}{c} \;\tau \frac{dV}{dt}=-g_{l}\left(V-E_{l}\right)-g_{e}\left(t\right)V,\\ \tau_{e}\frac{dg_{e}}{dt}=-g_{e}+I_{syn}\left(t\right) \end{array} \end{array}$$

*V* is the membrane potential and *g*_*e*_ is the conductance variable. *E*_*l*_ (set to −65 mV in this example) is the reversal potential and τ (20 ms) and τ_*e*_ (5 ms) represent the time scales for *V* and *g*_*e*_, respectively. *I*_*syn*_ represents changes to the conductance variable due to incoming spikes. If *V* is raised above a specified threshold value (−55 mV), it is reset to a specified reset membrane potential (−65 mV). The positions of individual neurons change in state space, both under the influence of the neuron's endogenous dynamics as determined by the dynamical system and of spike trains arriving from neurons in other populations, which cause rapid transitions in state space that are modeled as instantaneous jumps. For the simulation techniques mentioned earlier involving a large number of individual model neuron instances, a practice that we will refer to as Monte Carlo simulation, the population can be represented as a cloud of points in state space. The approach in MIIND, known as a population density technique (PDT), models the probability density of the cloud (shown in Figure 1 as a heat map) rather than the behaviour of individual neurons. The threshold and reset values of the underlying neuron model are visible in the hard vertical edges of the density in Figure 1A. In Figure 1B, the same simulation approach is used for a population of Fitzhugh-Nagumo neurons (FitzHugh, 1961; Nagumo et al., 1962). The dynamical system is defined in Equation (2).

(2) $$\begin{array}{l} \begin{array}{c} \frac{dV}{dt}=V-\frac{V^{3}}{3}-W,\\ \frac{dW}{dt}=0.08\left(V+0.7-0.8W\right) \end{array} \end{array}$$

*V* represents the membrane potential and *W* is a recovery variable. The Fitzhugh-Nagumo model has no threshold-reset mechanism and so there are no vertical boundaries to the density. As well as the density function being informative in itself, common population metrics such as average firing rate and average membrane potential can be quickly derived. The MIIND model archive, available in the code repository, contains example simulation files for populations of both conductance based neurons and Fitzhugh-Nagumo neurons (*examples/model_archive/Conductance2D* and *examples/model_archive/FitzhughNagumo*).

### 1.2. The Case for Population Density Techniques

Why use this technique? Nykamp and Tranchina (2000), Omurtag et al. (2000), Kamps (2003), Iyer et al. (2013) have demonstrated that PDTs are much faster than Monte Carlo simulation for 1D models; De Kamps et al. (2019) have shown that while speed is comparable between 2D models and Monte Carlo, memory usage is orders of magnitude lower because no spikes need to be buffered, which accounts for significant memory use in large-scale simulations. In practice, this may make the difference between running a simulation on an HPC cluster or a single PC equipped with a general purpose graphics processing unit (GPGPU).

Apart from simulation speed, PDTs have been important in understanding population level behaviour analytically. Important questions, such as “why are cortical networks stable?” (Amit and Brunel, 1997), “how can a population be oscillatory when its constituent neurons fire sporadically?” (Brunel and Hakim, 1999), “how does spike shape influence the transmission spectrum of a population?” (Fourcaud-Trocmé et al., 2003) have been analysed in the context of population density techniques, providing insights that cannot be obtained from merely running simulations. A particularly important question, which has not been answered in full is: “how do rate-based equations emerge from populations of spiking neurons and when is their use appropriate?” There are many situations where such rate-based equations are appropriate, but some where they are not and their correspondence to the underlying spiking neural dynamics is not always clear (de Kamps, 2013; Montbrió et al., 2015). There is a body of work suggesting that some rate-based equations can be seen as the lowest order of perturbations of a stationary state, and much of this work is PDT-based (Wilson and Cowan, 1972; Gerstner, 1998; Mattia and Del Giudice, 2002, 2004; Montbrió et al., 2015). MIIND opens the possibility to incorporate these theoretical insights into large-scale network models. For example, we can demonstrate the prediction from Brunel and Hakim (1999) that inhibitory feedback on a population can cause a bifurcation and produce resonance. Finally, for a steady state input, the firing rate prediction of a PDT model converges to a transfer function which can be used in artifical spiking neural networks (De Kamps et al., 2008).

### 1.3. Population-Level Modeling

For the population density approach we take with MIIND, the time evolution of the probability density function is described by a partial integro-differential equation. We give it here to highlight some of its features, but for an in depth introduction to the formalism and a derivation of the central equations we refer to Omurtag et al. (2000).

(3) $$\begin{array}{l} \frac{\partial \rho }{\partial t}+\frac{\partial }{\partial \vec{v}}\cdot \left(\frac{\vec{F}\left(\vec{v}\right)\rho \left(\vec{v},t\right)}{\tau }\right)=\int_{M}d{\vec{v}}^{\prime }\left\{W\left(\vec{v}|{\vec{v}}^{\prime }\right)\rho \left(\vec{v^{\prime }}\right)-W\left({\vec{v}}^{\prime }|\vec{v}\right)\rho \left(\vec{v}\right)\right\}, \end{array}$$

ρ is the probability density function defined over a volume of state space, *M*, in terms of time, *t*, and time-dependent variables, $\vec{v}$, under the assumption that the neuronal dynamics of a point model neuron is given by:

(4) $$\begin{array}{l} \tau \frac{d\vec{v}}{dt}=\vec{F}\left(\vec{v}\right), \end{array}$$

where τ is the neuron's membrane time constant. Simple models are one-dimensional (1D). For the leaky-integrate-and-fire (LIF) neuron:

(5) $$\begin{array}{l} F\left(v\right)=-v, \end{array}$$

For a quadratic-integrate-and-fire (QIF) neuron:

(6) $$\begin{array}{l} F\left(v\right)=v^{2}+I, \end{array}$$

where *v* is the membrane potential, and *I* can be interpreted as a bifurcation parameter. More complex models require a higher dimensional state space. Since such a space is hard to visualise and understand, considerable effort has been invested in the creation of effective models. In particular two-dimensional (2D) models are considered to be a compromise that allows considerably more biological realism than LIF or QIF neurons, but which remain amenable to visualisation and analysis, and can often be interpreted geometrically (Izhikevich, 2007). Examples are the Izhikevich simple neuron (Izhikevich, 2003), the Fitzhugh-Nagumo neuron (FitzHugh, 1961; Nagumo et al., 1962), and the adaptive-exponential-integrate-and-fire neuron (Brette and Gerstner, 2005), incorporating phenomena such as bursting, bifurcations, adaptation, and others that cannot be accounted for in a one dimensional model.

*W*(*v*∣*v*′) in Equation (3) represents a transition probability rate function. The right hand side of Equation (3) makes it a Master equation. Any Markovian process can be represented by a suitable choice of *W*. For example, for shot noise, we have

(7) $$\begin{array}{l} W\left(v^{\prime }|v\right)=\nu \left(\delta \left(v^{\prime }-v-h\right)-\delta \left(v-v^{\prime }\right)\right), \end{array}$$

where ν is the rate of the Poisson process generating spike events. The delta functions reflect that an incoming spike causes a rapid change in state space, modeled as an instantaneous jump, *h*. It depends on the particular neural model in what variable the jumps take place. Often models use a so-called delta synapse, such that the jump is in membrane potential. In conductance based models, the incoming spike causes a jump in the conductance variable (Figure 1A), and the influence of the incoming spike on the potential is then indirect, given by the dynamical system's response to the sudden change in the conductance state.

MIIND produces a numerical solution to Equation (3) for arbitrary 1D or 2D versions of $\vec{F}\left(\vec{v}\right)$ (support for 3D versions is in development), under a broad variety of noise processes. Indeed, the right hand side of Equation (3) can be generalised to other renewal processes which cannot simply be formulated in terms of a transition probability rate function *W*. It is possible to introduce a right hand side that entails an integration over a past history of the density using a kernel whose shape is determined by a Gamma distribution or other renewal process (Lai and de Kamps, 2017).

### 1.4. Quick Start Guide

Before describing the implementation details of MIIND, this section demonstrates how to quickly set up a simulation for a simple E-I network of populations of conductance based neurons using the MIIND Python library. A rudimentary level of Python experience is needed to run the simulation. In most cases, MIIND can be installed via Python pip. Detailed installation instructions can be found via the *README.md* file of the MIIND repository and in the MIIND documentation (Osborne and De Kamps, 2021). For this example, we will use a pre-written script, *generateCondFiles.py*, to generate the required simulation files which can be found in the *examples/quick_start* directory of the MIIND repository or can be loaded into a working directory using the following python command.


  $ python -m miind.loadExamples


In the *examples/quick_start* directory, the *generateCondFiles.py* script generates the simulation files, *cond.model* and *cond.tmat*.


  $ python generateCondFiles.py


The contents of *generateCondFiles.py* is given in Listing 1. The two important parts of the script are the neuron model function, in this case named *cond()*, and the call to the MIIND function *grid_generate.generate()* which takes a number of parameters which are discussed in detail later.

**Listing 1:**
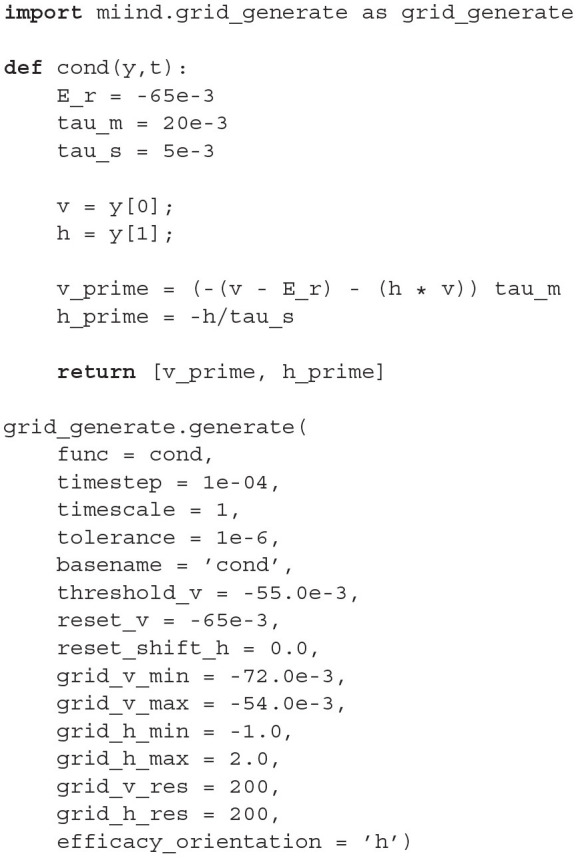
*generateCondFiles.py*.

The *cond()* function should be familiar to those who have used Python numerical integration frameworks such as *scipy.integrate*. It takes the two time dependent variables defined by *y*[0] and *y*[1] and a placeholder parameter, *t*, for performing a numerical integration. In the function, the user may define how the derivatives of each variable are to be calculated. The *generate()* function requires a suitable time step, values for a threshold and reset if needed, and a description of the extent of the state space to be simulated. With this structure, the user may define any two dimensional neuron model. The generated files are then referenced in a second file which describes a network of populations to be simulated. Listing 2 shows the contents of *cond.xml* describing an E-I network which uses the generated files from *generateCondFiles.py*.

**Listing 2:**
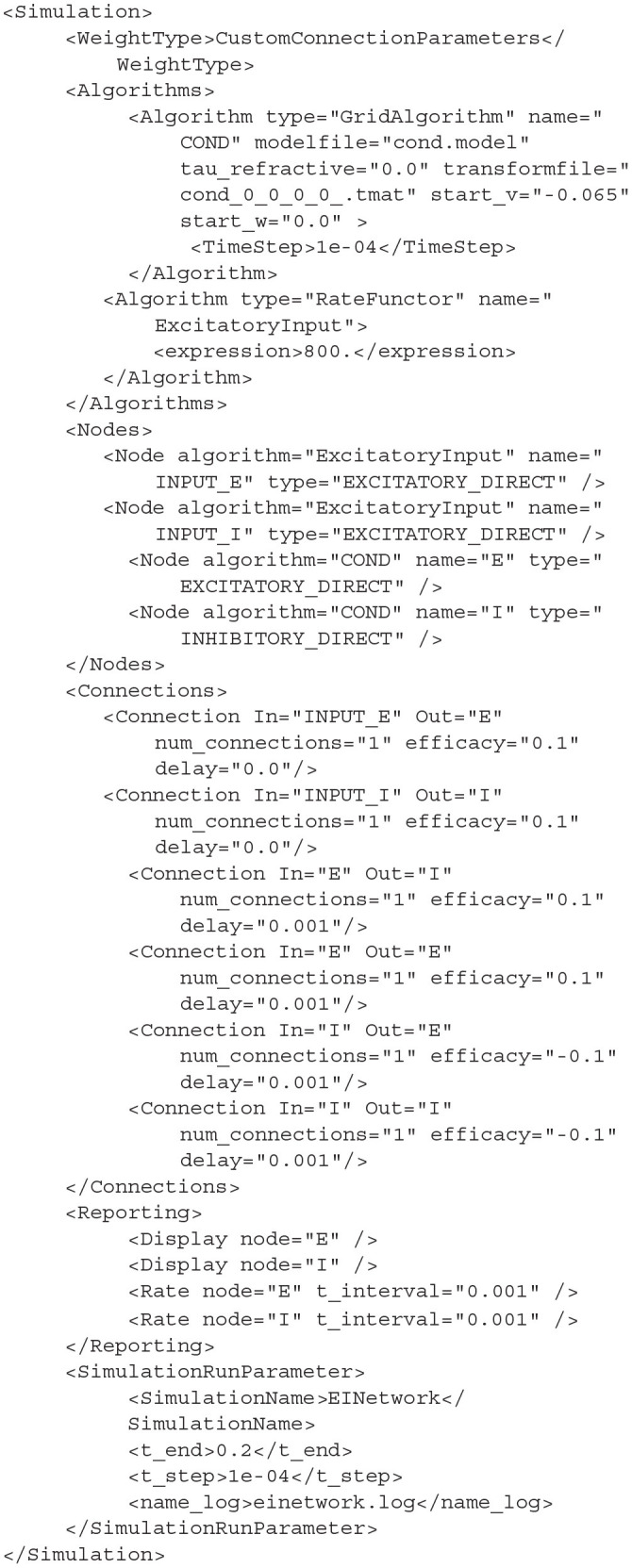
*cond.xml*.

The full syntax documentation for MIIND XML files is given in section 4. Though more compact or flexible formats are available, XML was chosen as a formatting style due to its ubiquity ensuring the majority of users will already be familiar with the syntax. The *Algorithms* section is used to declare specific simulation methods for one or more populations in the network. In this case, a GridAlgorithm named *COND* is set up which references the *cond.model* and *cond.tmat* files. A RateFunctor algorithm produces a constant firing rate. In the *Nodes* section, two instances of *COND* are created: one for the excitatory and inhibitory populations, respectively. Two *ExcitatoryInput* nodes are also defined. The *Connections* section allows us to connect the input nodes to the two conductance populations. The populations are connected to each other and to themselves with a 1ms transmission delay. The remaining sections are used to define how the output of the simulation is to be recorded, and to provide important simulation parameters such as the simulation time. By running the following python command, the simulation can be run.

**Listing 3:**
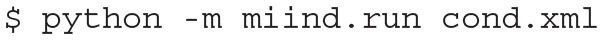
Run the cond.xml simulation.

**Figure 2:**
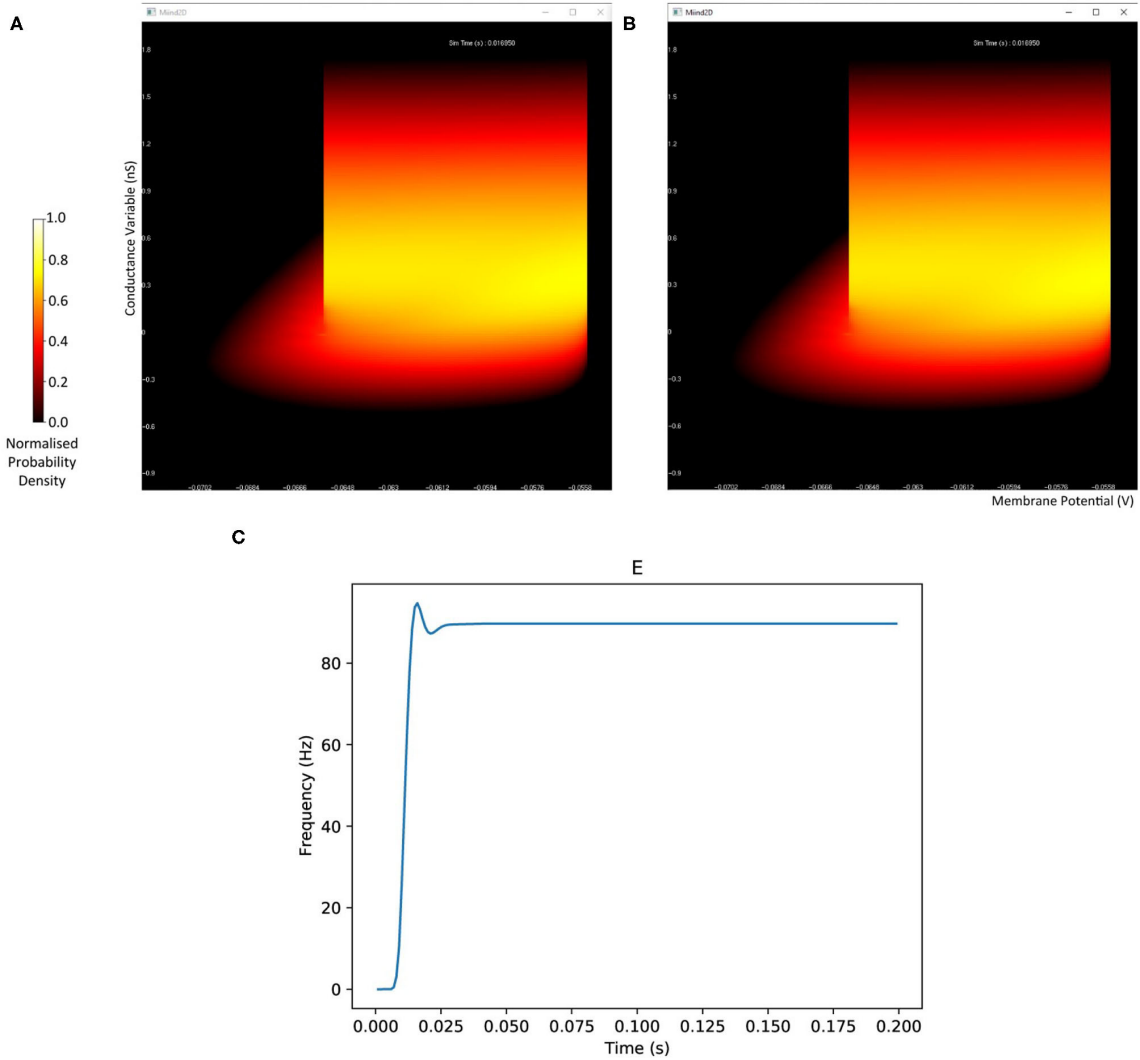
The display output of a running E-I population network simulation of conductance based neurons. **(A)** The probability density heat map (normalised to the maximum density value) of the excitatory population. **(B)** The probability density heat map of the inhibitory population. Brighter colours indicate a larger probability mass. The axes are unlabelled in the simulation windows as the software is agnostic to the underlying model. However, the membrane potential and conductance labels have been added for clarity. **(C)** The average firing rate of the excitatory population.

The probability density plots for both populations will be displayed in separate windows as the simulation progresses. The firing rate of the excitatory population can be plotted using the following commands. Figure 2 shows the probability density plots for both populations and average firing rate of population E.

**Listing 4:**
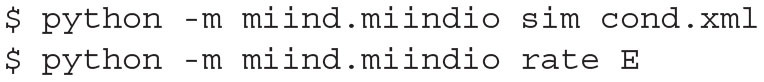
Load the cond.xml simulation and plot the average firing rate of population E.

Finally, the density function of each population can be plotted as a heat map for a given time in the simulation.

**Listing 5:**
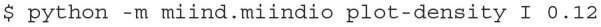
Plot the probability density of population I at time 0.12s.

Later sections will show how the MIIND simulation can be imported into a user defined Python script so that input can be dynamically set during simulation and population activity can be captured for further processing.

## 2. The MIIND Grid Algorithm

MIIND allows the user to simulate populations of any 1D or 2D neuron model. Although much of MIIND's architecture is agnostic to the integration technique used to simulate each population, the system is primarily designed to make use of its novel population density techniques, grid algorithm and mesh algorithm. Both algorithms use a discretisation of the underlying neuron model's state space such that each discrete “cell,” which covers a small area of state space, is considered to hold a uniform distribution of probability mass. In both algorithms, MIIND performs three important steps for each iteration. First, probability mass is transferred from each cell to one or more other cells according to the dynamics of the underlying neuron model in the absence of any input. The probability mass is then spread across multiple other cells due to incoming random spikes. Finally, if the underlying neuron model has a threshold-reset mechanic, such as an integrate and fire model, probability mass which has passed the threshold is transferred to cells along the reset potential. As it is the most practically convenient method for the user, we will first introduce the grid algorithm. We will discuss its benefits and weaknesses, indicating where it may be appropriate to use the mesh algorithm instead.

### 2.1. Generating the Grid and Transition Matrix

**Figure 3:**
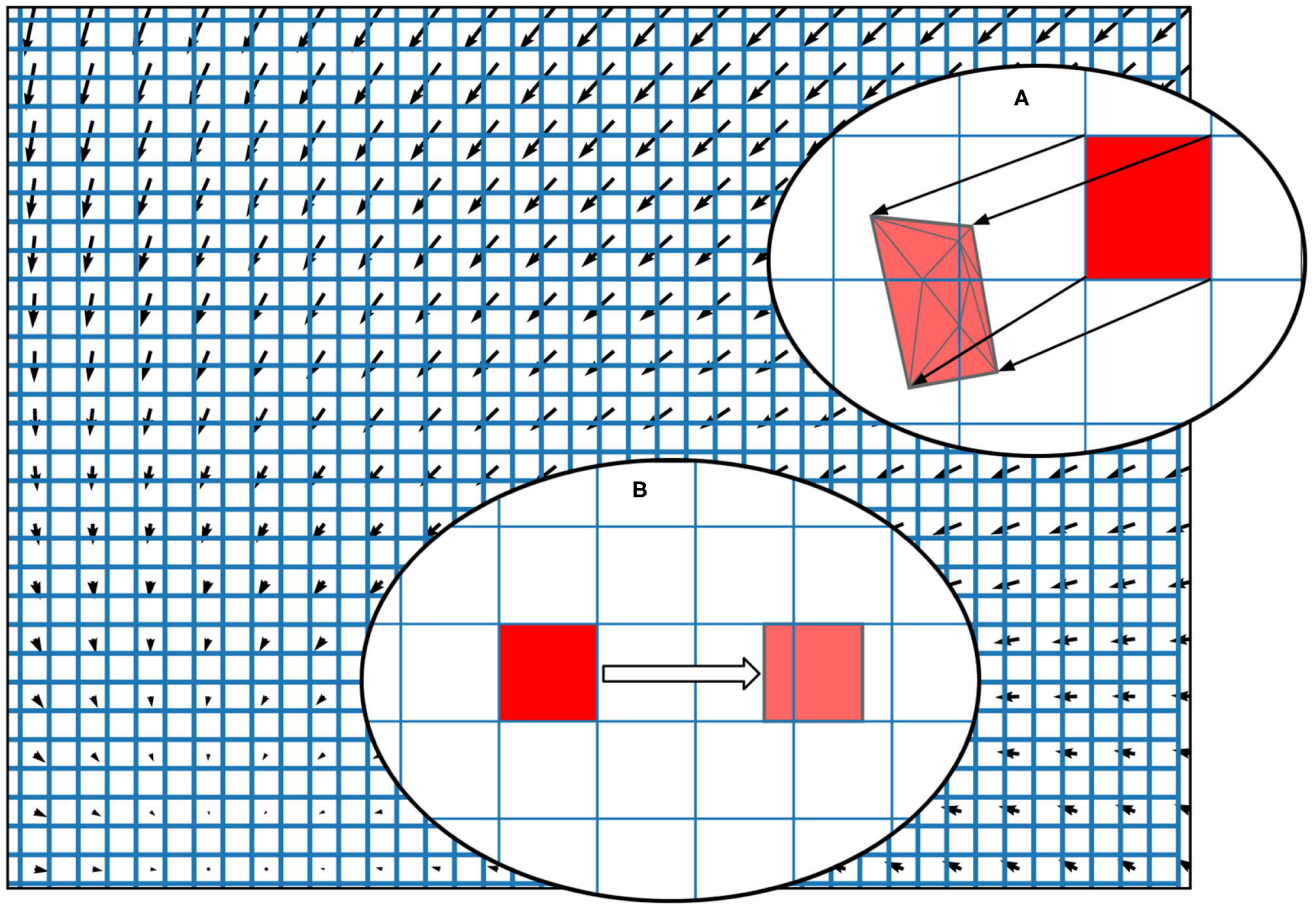
The state space of a neuron model (shown here as a vector field) is discretised into a regular grid of cells. **(A)** The transition matrix for solving the deterministic dynamics of the population is generated by applying a single time step of the underlying neuron model to each vertex of each cell in the grid and calculating the proportion by area to each overlapping cell. Once the vertices of a grid cell have been translated, the resulting polygon is recursively triangulated according to intersections with the original grid. Once complete, all triangles can be assigned to a cell and the area proportions can be summed. **(B)** For a single incoming spike (with constant efficacy), all cells are translated by the same amount and therefore have the same resulting transition which can be used to solve the Poisson master equation. In fact, the transition will always involve at most two target cells and the proportions can be calculated knowing only the grid cell width and the efficacy.

To discretise the state space in the grid method, the user can specify the size and *M* × *N* resolution of a rectangular grid which results in *MN* identical rectangular cells, each of which will hold probability mass. In the grid algorithm, a transition matrix lists the proportion of mass which moves from each cell to (usually) adjacent cells in one time step due to the deterministic dynamics of the underlying neural model. To pre-calculate the transitions for each cell, MIIND first translates the vertices of every cell by integrating each point forward by one time step according to the dynamics of the underlying neuron model as shown in Figure 3A. As the time step is small, a single Euler step is usually all that is required to avoid large errors (although other integration schemes can be used if required). Each transformed cell is no longer guaranteed to be a rectangle and is compared to the original non-transformed grid to ascertain which cells overlap with the newly generated quadrilateral. An overlap indicates that some proportion of neurons in the original cell will move to the overlapping cell after one time step. In order to calculate the overlap, the algorithm in Listing 6 is employed. This algorithm is also used in the geometric method of generating transition matrices for the mesh algorithm shown later.

**Listing 6:**
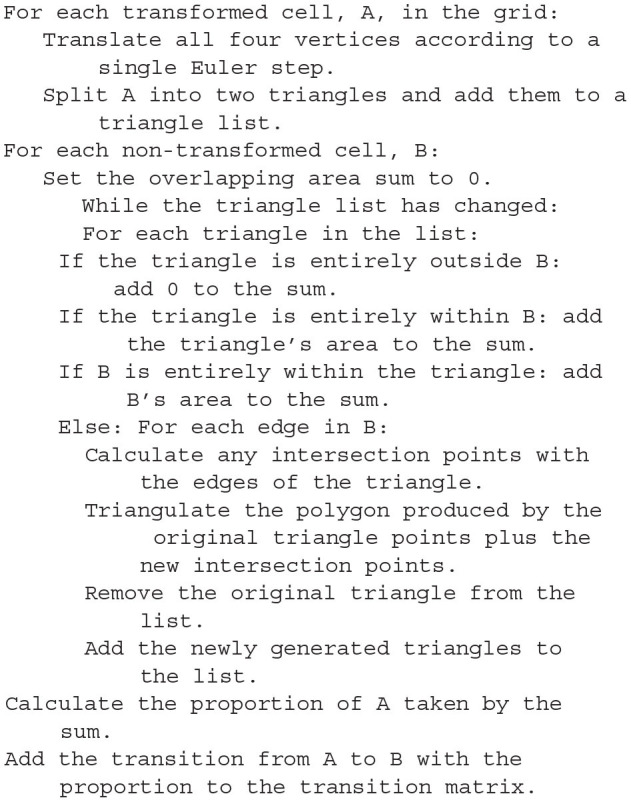
A pseudo-code representation of the algorithm used to calculate the overlapping areas between transformed grid cells and the original grid (or for translated cells of a mesh). The proportion of the area of the original cell gives the proportion of probability mass to be moved in each transition.

Though the pseudo-code algorithm is order *N*^2^, there are many ways that the efficiency of the algorithm is improved in the implementation. The number of non-transformed cells checked for overlap can be limited to only those which lie underneath each given triangle. Furthermore, the outer loop is parallelisable. Finally, as the non-transformed cells are axis-aligned rectangles, the calculation to find edge intersections is trivial. Figure 3A shows a fully translated and triangulated cell at the end of the algorithm. Once the transition matrix has been generated, it is stored in a file with the extension *.tmat*. Although the regular grid can be described with only four parameters (the width, height, X, and Y resolutions), to more closely match the behaviour of mesh algorithm, the vertices of the grid are stored in a *.model* file. To simulate a population using the grid algorithm, the *.tmat* and *.model* files must be generated and referenced in the XML simulation file.

As demonstrated in the quick start guide (section 1.4), to generate a *.model* and *.tmat* file, the user must write a short Python script which defines the underlying neuron model and makes a call to the MIIND API to run the algorithm in listing 6. In the *python* directory of the MIIND source repository (see Supplementary Section 1 in the Supplementary Material), there are a number of examples of these short scripts. The script used to generate a grid for the Izhikevich simple model is listed in the Supplementary Section 9.1. The required definition of the neuron model function is similar to those used by many numerical integration libraries. The function takes a parameter, *y*, which represents a list which holds the two time dependent variables and a parameter, *t*, which is a placeholder for use in integration. The function must return the first time derivatives of each variable as a list in the same order as in *y*. Once the function has been written, a call to *grid_generate.generate* is made which takes the parameters listed in Table 1.

**Table 1:** Parameters for the *grid_generate.generate* function.

**Parameter name**	**Notes**
*func*	The underlying neuron model function.
*timestep*	The desired time step for the neuron model
*timescale*	A scale factor for the timescale of the underlying neuron model to convert the time step into seconds.
*tolerance*	An error tolerance for solving a single time step of the neuron model.
*basename*	The base name with which all output files will be named.
*threshold_v*	The spike threshold value for integrate and fire neuron models.
*reset_v*	The reset value for integrate and fire neuron models.
*reset_shift_h*	A value for increasing the second variable during reset for integrate and fire neuron models with some adaptive shift or similar function.
*grid_v_min*	The minimum value for the first dimension of the grid (usually membrane potential).
*grid_v_max*	The maximum value for the first dimension of the grid.
*grid_h_min*	The minimum value for the second dimension of the grid.
*grid_h_max*	The maximum value for the second dimension of the grid.
*grid_v_res*	The number of columns in the grid.
*grid_h_res*	The number of rows in the grid.
*efficacy_orientation*	The direction, “v” or “h,” in which incoming spikes cause an instantaneous change.

When the user runs the script, the required *.model* and *.tmat* files will be generated for use in a simulation. In the quick start guide, the conductance based neuron model requires that *efficacy_orientation* is set to “h” because incoming spikes cause an instantaneous change in the conductance variable instead of the membrane potential. By default, however, this parameter is set to “v.” When choosing values for the grid bounds (*grid_v_min, grid_v_max, grid_h_min*, and *grid_h_max*), the aim is to estimate where in state space the population density function might be non-zero during a simulation. In the conductance based neuron model, because of the threshold-reset mechanic, the *grid_v_max* parameter need only be slightly above the threshold to ensure that there is at least one column of cells on or above threshold to allow probability mass to be reset. The *grid_v_min* value should be below the resting potential and reset potential. However, we must also consider that the neurons could receive inhibitory spikes which would cause the neurons to hyperpolarise. *grid_v_min* should therefore be set to a value beyond the lowest membrane potential expected during the simulation. Similarly for the conductance variable, space should be provided for reasonable positive and negative values. If it is known beforehand that no inhibition will occur, however, then the state space bounds can be set tighter in order to improve the accuracy of the simulation using the same grid resolution (*grid_v_res* and *grid_h_res*). If, during the simulation, probability mass is pushed beyond the lower bounds of the grid, it will be pinned at those lower bounds which will produce incorrect behaviour and results. If the probability mass is pushed beyond the upper bounds, it will be wrapped around to the lower bounds which will also produce incorrect results. The choice of grid resolution is a balance between speed of simulation and accuracy. However, even very coarse grids can produce representative firing rates and behaviours. Typical grid resolutions range between 100 × 100 and 500 × 500. It can also be beneficial to experiment with different *M* and *N* values as the accuracy of each dimension can have unbalanced influence over the population level metrics.

### 2.2. The Effect of Random Incoming Spikes

The transition matrix in the *.tmat* file describes how probability mass moves to other cells due to the deterministic dynamics of the underlying neuron model. The transition matrix is sparse as probability mass is often only transferred to nearby cells. Solving the deterministic dynamics is therefore very efficient. The mesh algorithm is even faster and, as demonstrated later, is significantly quicker than direct simulation for this part of the algorithm. Another benefit to the modeler is that by rendering the grid with each cell coloured according to its mass, the resultant heat map gives an excellent visualisation of the state of the population as a whole at each time step of the simulation as shown in Figure 2. This provides particularly useful insight into the sub-threshold behaviour of neurons in the population.

The second step of the grid algorithm, which must be performed every iteration, is to solve the change in the probability density function due to random incoming spikes. It is assumed that a spike causes an instantaneous change in the state of a neuron, usually a step wise jump in membrane potential corresponding to a constant synaptic efficacy. In the conductance based neuron example, this jump is in the conductance. When considering each cell in the grid, a single incoming spike will cause some proportion of the probability mass to shift to at most, two other cells as shown in Figure 3. Because all cells in the grid are equally distributed and the same size, the relative transition of probability mass caused by a single spike is the same for them all. A sparse transition matrix, *M*, can be generated from this single transition so that applying *M* to the probability density grid applies the transition to all cells. MIIND calculates a different *M* for each incoming connection to the population based on the user defined instantaneous jump, which we refer to as the efficacy. In the mesh algorithm, the relative transitions are different for each cell and so a transition matrix (similar to that of the *.tmat* file) is required to describe the effect of a single spike. As with many other population density techniques, MIIND assumes that incoming spikes are Poisson distributed, although it is possible to approximate other distributions. MIIND uses *M* to calculate the change to the probability density function, ρ, due solely to the non-deterministic dynamics as described by Equation 8.

(8) $$\begin{array}{l} d\rho /dt=\lambda M\rho \end{array}$$

λ is the incoming Poisson firing rate. The boost numeric library is used to integrate *dρ*/*dt*. The solution to this equation describes the spread of the probability density due to Poisson spikes. This “master process” step amounts to multiple applications of the transition matrix *M* and is where the majority of time is taken computationally. However, OpenMP is available in MIIND to parallelise the matrix multiplication. If multiple cores are available, the OpenMP implementation significantly improves performance of the master process step. More information covering this technique can be found in de Kamps (2013), De Kamps et al. (2019).

### 2.3. Threshold-Reset Dynamics

**Figure 4:**
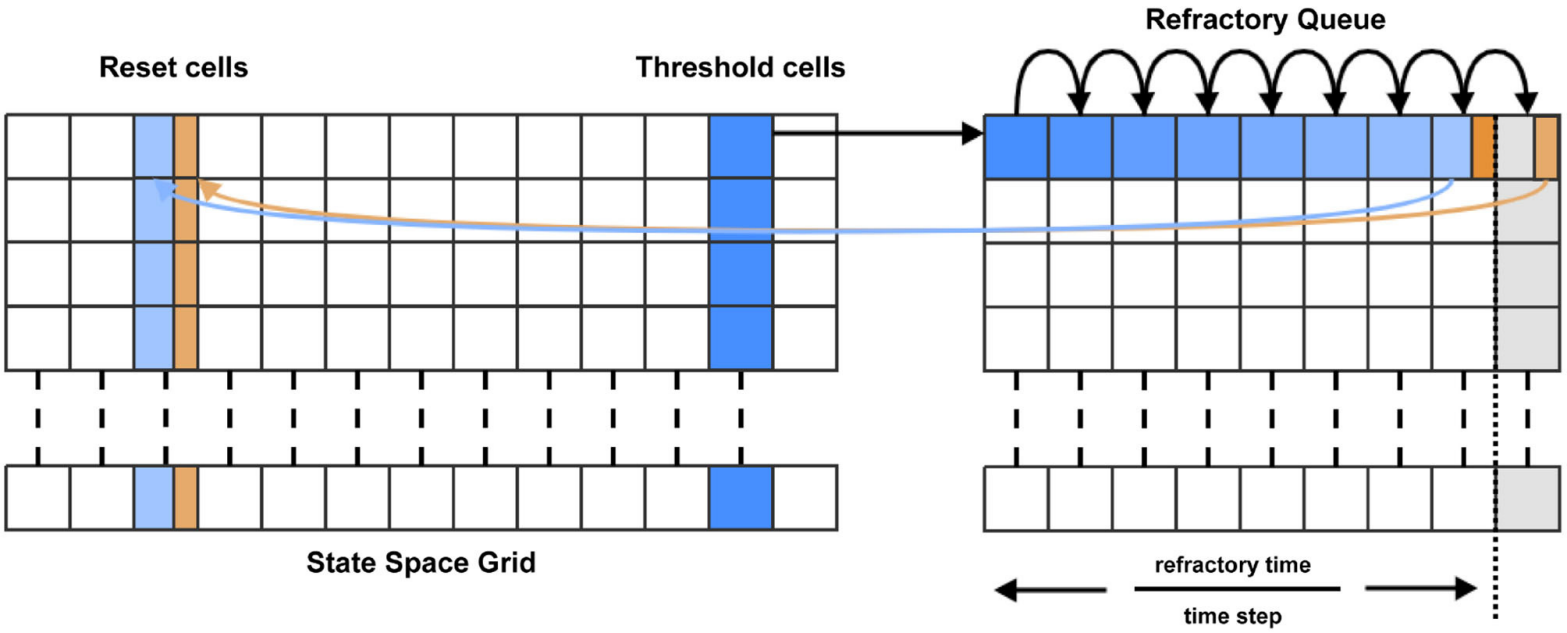
For each time step, probability mass in the cells which lie across the threshold (threshold cells) is pushed onto the beginning of the refractory queue. There is one queue per threshold cell. During each subsequent time step, the probability mass is shifted one place along the queue until it reaches the penultimate place. A proportion of the mass, calculated according to the modulo of the refractory time and the time step, is transferred to the appropriate reset cell. The remaining mass is shifted to the final place in the queue. During the next time step, that remaining mass is transferred to the reset cell.

Many neuron models include a “threshold-reset” process such that neurons which pass a certain membrane potential value are shifted back to a defined reset potential to approximate repolarisation during an action potential. To facilitate this in MIIND, after each iteration, probability mass in cells which lie across the threshold potential is relocated to cells which lie across the reset potential according to a pre-calculated mapping. Often, a refractory period is used to hold neurons at the reset potential before allowing them to again receive incoming spikes. In MIIND this is implemented using a queue for each threshold cell as shown in Figure 4. The queues are set to the length of the refractory period divided by the time step, rounded up to the nearest integer value. During each iteration, probability mass is shifted one position along the queue. A linear interpolation of the final two places in the queue is made and this value is passed to the mapped reset cell. The interpolation is required in case the refractory period is not an integer multiple of the time step. The total probability mass in the threshold cells each iteration is used to calculate the average population firing rate. For models which do not require threshold-reset dynamics, setting the threshold value to the maximal membrane potential of the grid, and the reset to the minimal membrane potential ensures that no resetting of probability mass will occur.

### 2.4. How MIIND Facilitates Interacting Populations

The grid algorithm describes how the behaviour of a single population is simulated. The MIIND software platform as a whole provides a way for many populations with possibly many different integration algorithms to interact in a network. The basic process of simulating a network is as follows. The user must write an XML file which describes the whole simulation. This includes defining the population nodes of the network and how they are connected; which integration technique each population uses (grid algorithm, mesh algorithm etc.); external inputs to the network; how the activity of each population will be recorded and displayed; the length and time step of the simulation. As shown in the quick start guide, the XML file can be passed as a parameter to the *miind.run* module in Python. When the simulation is run, a population network is instantiated and the simulation loop is started. For each iteration, the output activity of each population node is recorded. By default, the activity is assumed to be an average firing rate but other options are available such as average membrane potential. The outputs are passed as inputs to each population node according to the connectivity defined in the XML file. Each population is evolved forward by one time step and the simulation loop repeats until the simulation time is up. The Python front end, *miind.miindio*, provides the user with tools to analyse the output from the simulation. A custom *run* script can also be written by the user to perform further analysis and processing.

**Figure 5:**
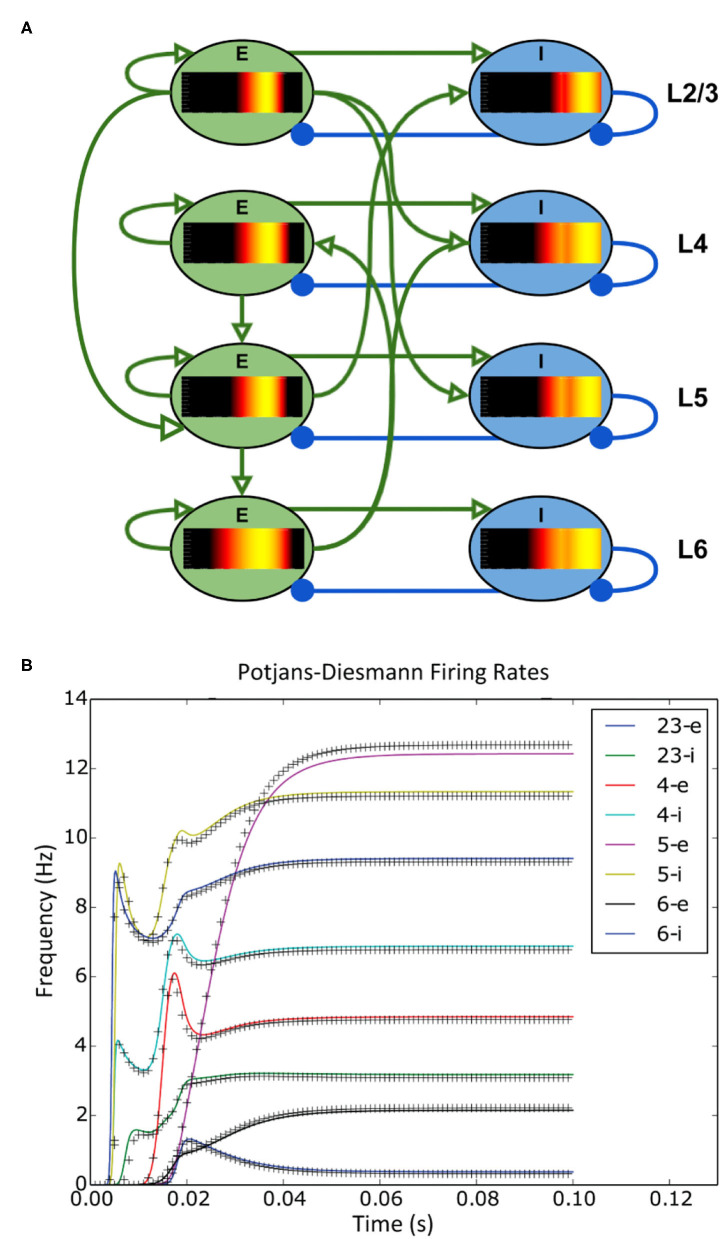
**(A)** A representation of the connectivity between populations in the Potjans-Diesmann microcircuit model. Each population shows the probability density at an early point in the simulation before all populations have reached a steady state. All populations are of leaky-integrate-and-fire neurons and so the density plots show membrane potential in the horizontal axis. The vertical axis has no meaning (probability mass values are the same at all points along the vertical). **(B)** The firing rate outputs from MIIND (crosses) in comparison to those from DiPDE for the same model (solid lines).

The simplicity of the XML file means that a user can set up a large network of populations with very little effort. The model archive in the code repository holds a set of example simulations demonstrating the range of MIIND's functionality and includes an example which simulates the Potjans-Diesmann model of a cortical microcircuit (Potjans and Diesmann, 2014), which is made up of eight populations of leaky integrate and fire neurons. Figure 5 shows a representation of the model with embedded density plots for each population.

### 2.5. Running MIIND Simulations

The quick start guide demonstrated the simplest way to run a simulation given that the required *.model, .tmat*, and *.XML* files have been generated. The *miind.run* script imports the *miind.miindsim* Python extension module which can also be imported into any user written Python script. Section 6 details the functions which are exposed by *miind.miindsim* for use in a python script. The benefit of this method is that the outputs from populations can be recorded after each iteration and inputs can be dynamic allowing the python script to perform its own logic on the simulation based on the current state.

There is also a command line interface (CLI) program provided by the Python module, *miind.miindio*. The CLI can be used for many simple work flow tasks such as generating models and displaying results. Each command which is available in the CLI, can also be called from the MIIND Python API, upon which the CLI is built. A full list of the available commands in the CLI is given in section 9.3 of the Supplementary Material and a worked example using common CLI commands is provided in section 7.

### 2.6. When Not to Use the Grid Algorithm

**Figure 6:**
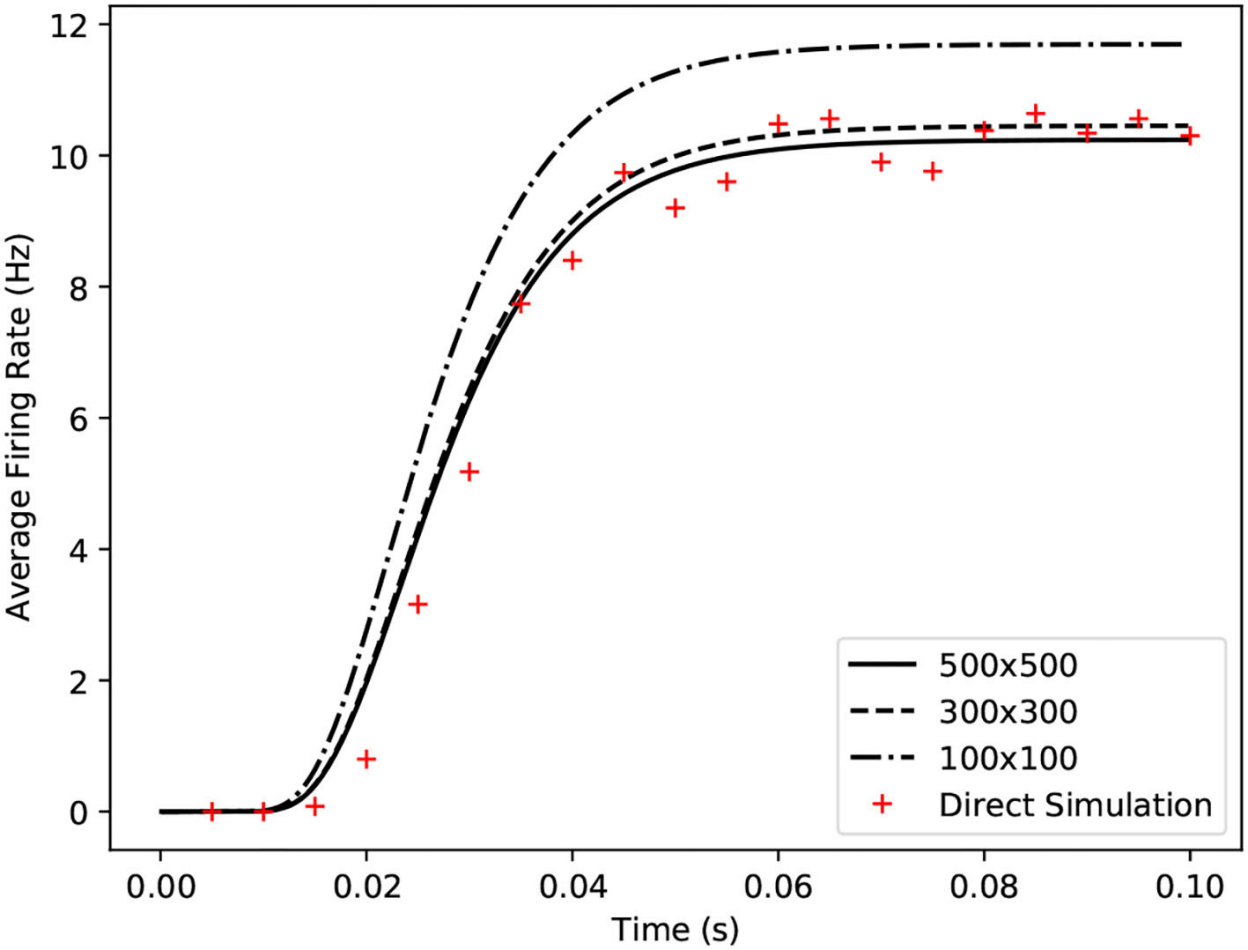
Comparison of average firing rates from four simulations of a single population of conductance based neurons. The black solid and dashed lines indicate MIIND simulations using the grid algorithm with different grid resolutions. The red crosses show the average firing rate of a direct simulation of 10,000 neurons.

**Figure 7:**
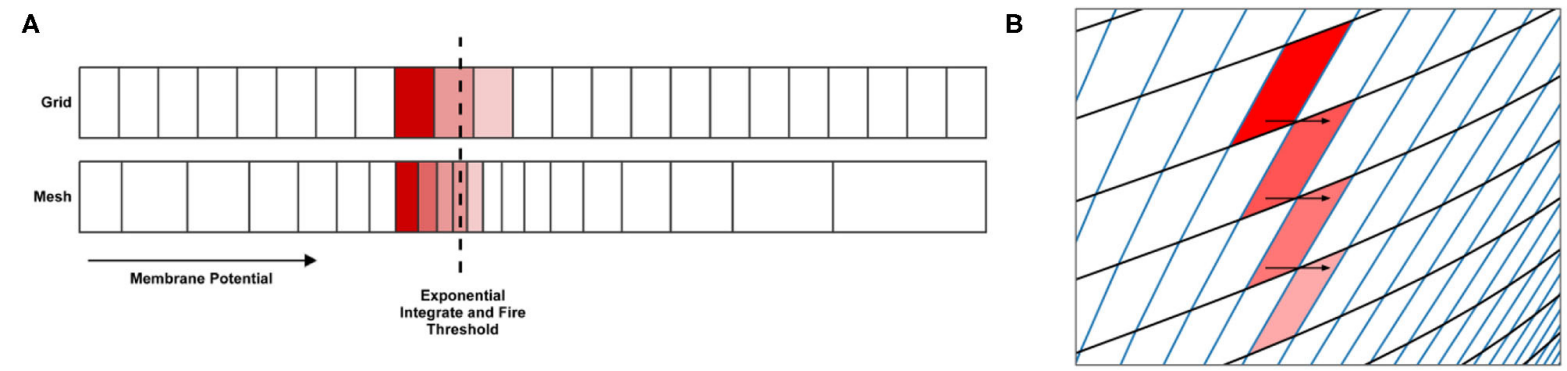
**(A)** In the grid algorithm, large cells cause probability mass to be distributed further than it should. This error is expressed most clearly in models where the average firing rate of the population is highly dependent on the amount of probability mass passing through an area of slow dynamics. **(B)** In the mesh algorithm, when cells become shear, probability mass which is pushed to the right due to incoming spikes also moves laterally (downwards) because it is spread evenly across each cell.

For many underlying neuron models, the grid algorithm will produce results showing good agreement with direct simulation to a greater or lesser extent depending on the resolution of the grid (see Figure 6). However, for models such as exponential integrate and fire, a significantly higher grid resolution is required than might be expected because of the speed of the dynamics across the threshold (beyond which, neurons perform the action potential). When the input rate is high enough to generate tonic spiking in an exponential integrate and fire model, the rate of depolarisation of each neuron reduces as it approaches the threshold potential then once it is beyond the threshold, quickly increases producing a spike. Because the grid discretises the state space into regular cells, if cells are large due to a low resolution, only a small number of cells will span the threshold, as shown in Figure 7A. When the transition matrix is applied each time step, probability mass is distributed uniformly across each cell. Probability mass can therefore artificially cross the threshold much faster than it should leading to a higher than expected average firing rate for the population. Using the grid algorithm for such models where the firing rate itself is dependent on sharp changes in the speed of the dynamics should be avoided if high accuracy is required. Other neuron models, like the busting Izhikevich simple model, also have sharp changes in speed when neurons transition from bursting to quiescent periods. However, the bursting firing rate is unaffected by these dynamics and the oscillation frequency is affected only negligibly due to the difference in timescales. The grid algorithm is therefore still appropriate in cases such as this. For exponential integrate and fire models, however, MIIND provides a second algorithm which can more accurately capture the deterministic dynamics: mesh algorithm.

## 3. The MIIND Mesh Algorithm

**Figure 8:**
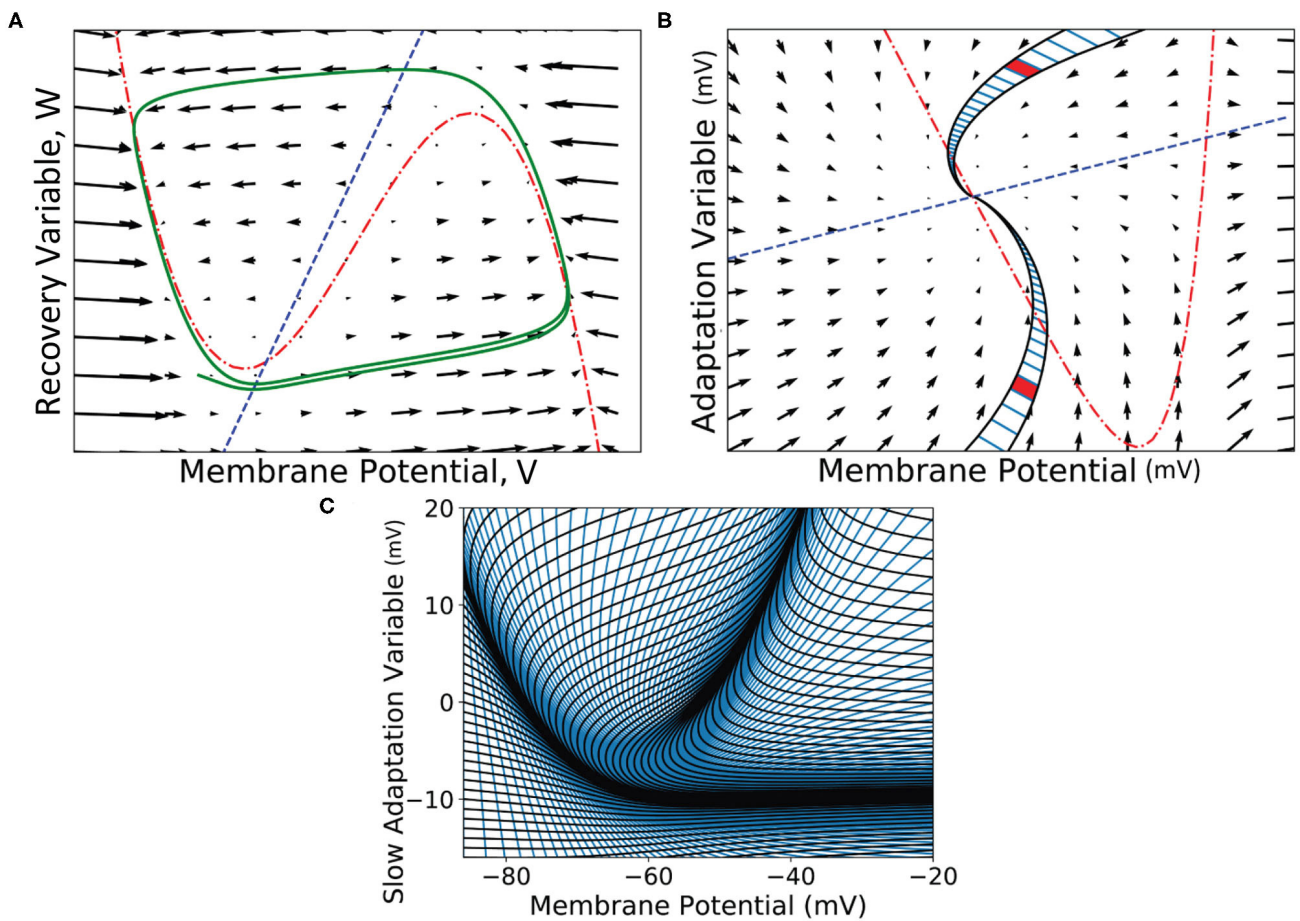
**(A)** A vector field of the FitzHugh-Nagumo neuron model (FitzHugh, 1961). Arrows show the direction of motion of states through the field according to the dynamics of the model. The red broken dashed nullcline indicates where the change in *V* is zero. The blue dashed nullcline indicates where the change in *W* is zero. The green solid line shows a potential path (trajectory) of a neuron in the state space. **(B)** A vector field for the adaptive exponential integrate and fire neuron model (Brette and Gerstner, 2005). Two strips are shown which follow the dynamics of the model and approach the stationary point where the nullclines cross. A strip is constructed between two trajectories in state space. Each time step of the two trajectories is used to segment the strip into cells. Because the strips approach a stationary point, they get thinner as the trajectories converge to the same point and cells get closer together as the distance in state space travelled reduces per time step (neurons slow down as they approach a stationary point). Per time step, probability mass is shifted from one cell to the next along the strip. **(C)** The state space of the Izhikevich simple neuron model (Izhikevich, 2003) which has been fully discretised into strips and cells.

Instead of a regular grid to discretise the state space of the underlying neuron model, the mesh algorithm requires a two dimensional mesh which describes the dynamics of the neuron model itself in the absence of incoming spikes. A mesh is constructed from strips which follow the trajectories of neurons in state space (Figure 8). The trajectories form so-called characteristic curves of the neuron model from which this method is inspired (de Kamps, 2013; De Kamps et al., 2019).

These trajectories are computed as part of a one-time preprocessing step using an appropriate integration technique and time step. Strips will often approach or recede from nullclines and stationary points and their width may shrink or expand according to their proximity to such elements. Each strip is split into cells. Each cell represents how far along the strip neurons will move in a single time step. As with the width of the strips, cells will become more dense or more sparse as the dynamics slow down and speed up, respectively. The result of covering the state space with strips is a precomputed description of the model dynamics such that the state of a neuron in one cell of the mesh is guaranteed to be in the next cell along the strip after a single time step. Depending on the underlying neuron model, it can be difficult to get full coverage without cells becoming too small or shear. However, once built, the deterministic dynamics have effectively been “pre-solved” and baked into the mesh.

As with the grid algorithm, when the simulation is running, each cell is associated with a probability mass value which represents the probability of finding a neuron from the population with a state in that cell. When a probability density function (PDF) is defined across the mesh, computing the change to the PDF due to the deterministic dynamics of the neurons is simply a matter of shifting each cell's probability mass value along its strip. In the C++ implementation, this requires no more than a pointer update and is therefore quicker than the grid algorithm for solving the deterministic dynamics as no transition matrix is applied to the cells.

**Figure 9:**
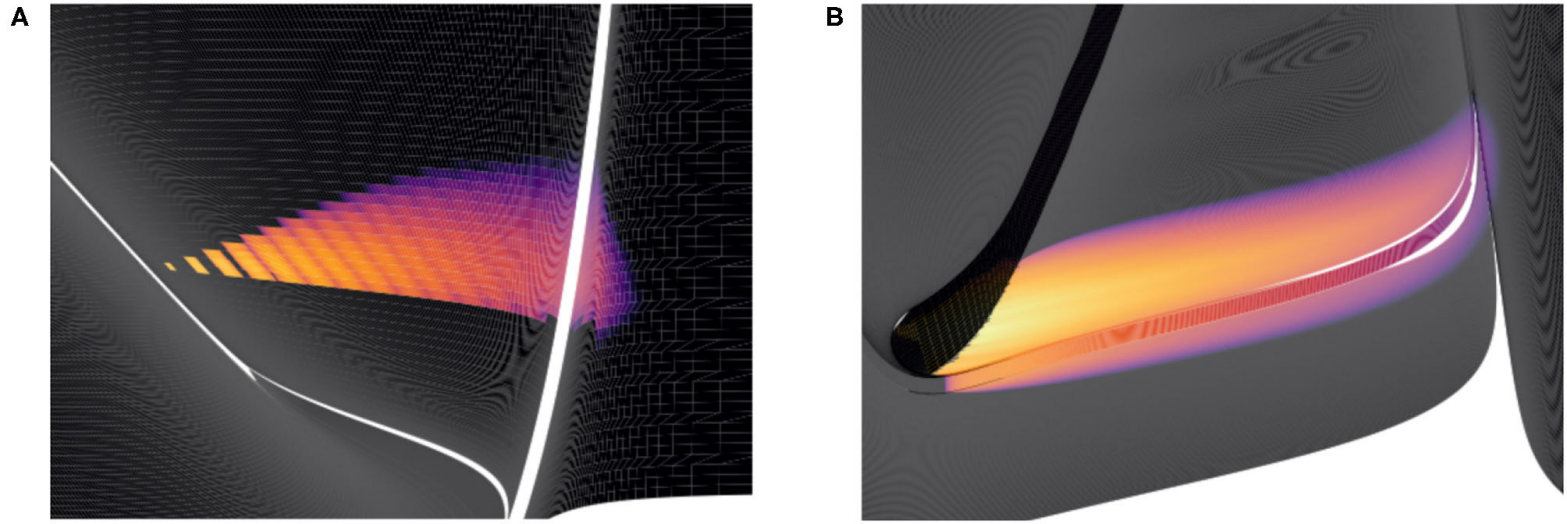
Heat plots for the probability density functions of two populations in MIIND. Brightness (more yellow) indicates a higher probability mass. Scales have been omitted as the underlying neuron models are arbitrary. **(A)** When the Poisson master equation is solved, probability mass is pushed to the right (higher membrane potential) in discrete steps. As time passes, the discrete steps are smoothed out due to the movement of mass according to the deterministic dynamics (following the strip). **(B)** A combination of mass travelling along strips and being spread across the state space by noisy input produces the behaviour of the population.

Mesh algorithm does, however, still require a transition matrix to implement the effect of incoming spikes on the PDF. This transition matrix describes how the state of neurons in each cell are translated in the event of a single incoming spike. Unlike the grid algorithm, cells are unevenly distributed across the mesh and are different sizes and shapes. What proportion of probability mass is transferred to which cells with a single incoming spike is, therefore, different for all cells. During simulation, the total change in the PDF is calculated by shifting probability mass one cell down each strip and using the transition matrix to solve the master equation every time step. The combined effect can be seen in Figure 9. The method of solving the master equation is explained in detail in de Kamps (2013).

### 3.1. When Not to Use the Mesh Algorithm

Just as with the grid algorithm, certain neuron models are better suited to an alternative algorithm. In the mesh algorithm, very little error is introduced for the deterministic dynamics. Probability mass flows down each strip as it would without the discretisation and error is limited only to the size of the cells. When the master equation is solved, however, probability mass can spread to parts of state space which would see less or no mass. Figure 7B demonstrates how in the mesh algorithm, as probability mass is pushed horizontally, very shear cells can allow mass to be incorrectly transferred vertically as well. In the grid algorithm, error is introduced in the opposite way. Solving the master equation pushes probability mass along horizontal rows of the grid and error is limited to the width of the row. The grid algorithm is preferable over the mesh algorithm for populations of neurons with one fast variable and one slow variable which can produce very shear cells in a mesh, e.g., in the Fitzhugh-Nagumo model (De Kamps et al., 2019). In both algorithms, the error can be reduced by increasing the density of cells (by increasing the resolution of the grid, or by reducing the timestep and strip width of the mesh). However, better efficiency is achieved by using the appropriate algorithm.

### 3.2. Building a Mesh for the Mesh Algorithm

**Figure 10:**
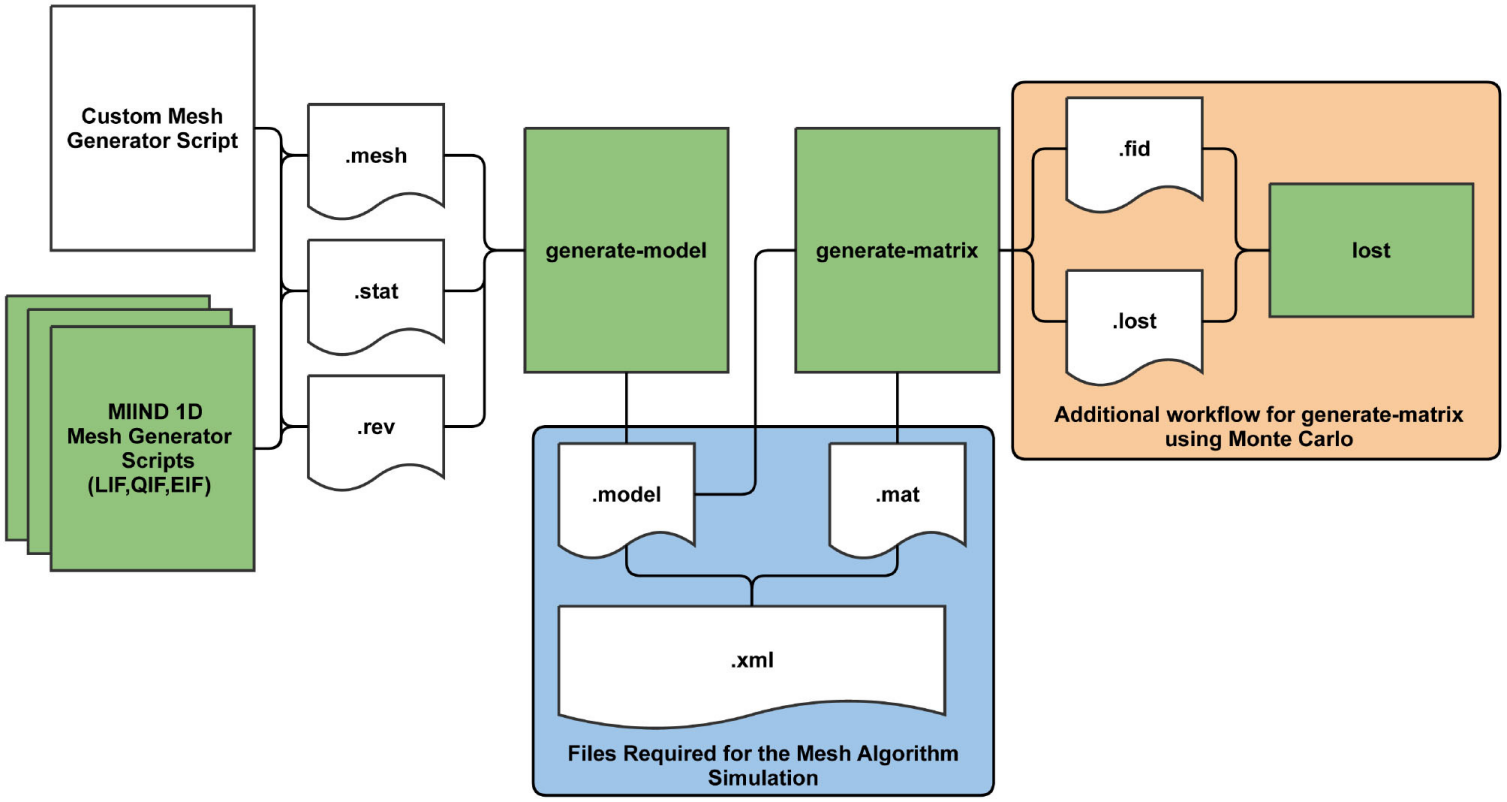
The MIIND processes and generated files required at each stage of pre-processing for the mesh algorithm. The shaded green rectangles represent automated processes run *via* the MIIND CLI.

Before a simulation can be run for a population which uses the mesh algorithm, the pre-calculation steps of generating a mesh and transition matrices must be performed. Figure 10 shows the full pre-processing pipeline for mesh algorithm. The mesh is a collection of strips made up of quadrilateral cells. As mentioned earlier, probability mass moves along a strip from one cell to the next each time step which describes the deterministic dynamics of the model. Defining the cells and strips of a 2D mesh is not generally a fully automated process and the points of each quadrilateral must be defined by the mesh developer and stored in a .*mesh* file. When creating the mesh, the aim is to cover as much of the state space as possible without allowing cells to get too small or misshapen. An example of a full mesh generation script for the Izhikevich simple neuron model (Izhikevich, 2003) is available in Supplementary Section 9.1 of the Supplementary Material. MIIND provides *miind.miind_api.LifMeshGenerator, miind.miind_api.QifMeshGenerator*, and *miind.miind_api.EifMeshGenerator* scripts to automatically build the 1D leaky integrate and fire, quadratic integrate and fire, and exponential integrate and fire neuron meshes, respectively. They can be called from the CLI. The scripts generate the three output files which any mesh generator script must produce: a *.mesh* file, a *.stat* file which defines extra cells in the mesh to hold probability mass that has settled at a stationary point, and a *.rev* file which defines a “reversal mapping” indicating how probability mass is transferred from strips in the mesh to the stationary cells. More information on *.mesh, .stat*, and *.rev* files is provided in the Supplementary Section 6.

Once the *.mesh, .stat*, and *.rev* files have been generated by the user or by one of the automated 1D scripts, the Python command line interface, *miind.miindio*, provides commands to convert the three files into a single *.model* file and generate transition matrices stored in *.mat* files. The model file is what will be referenced and read by MIIND to load a mesh for a simulation. To generate this file, use the CLI command, **generate-model**. The command parameters are shown in Table 2. All input files must have the same base name, for example: *lif.mesh, lif.stat*, and *lif.rev*. If the command runs successfully, a new file will be created: *basename.model*. A number of pre-generated models are available in the *examples* directory of the MIIND repository to be used “out of the box” including the adaptive exponential integrate and fire and conductance based neuron models.

**Table 2:** Parameters for the **generate-model** command in the CLI.

**Parameter name**	**Notes**
*basename*	The shared name of the *.mesh, .stat, .rev* and generated *.model* files.
*reset*	The value (usually representing membrane potential) which probability mass will be transferred to having passed the threshold.
*threshold*	The value (usually representing membrane potential) beyond which probability mass will be transferred to the reset value.

**Listing 7:**
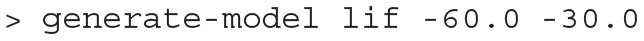
Generate a Model in the CLI

The generated *.model* file contains the mesh vertices, some summary information such as the time step used to generate the mesh and the threshold and reset values, and a mapping of threshold cells to reset cells.

In the mesh algorithm, transition matrices are used to solve the Poisson master equation which describes the movement of probability mass due to incoming random spikes. In the mesh algorithm, one transition matrix is required for each post synaptic efficacy that will be needed in the simulation. So if a population is going to receive spikes which cause jumps of 0.1 and 0.5 mV, two transition matrices are required. It is demonstrated later how the efficacy can be made dependent on the membrane potential or other variables. Each transition matrix is stored in a *.mat* file and contains a list of source cells, target cells, and proportions of probability mass to be transferred to each. For a given cell in the mesh, neurons with a state inside that cell which receive a single external spike will shift their location in state space by the value of the efficacy. Neurons from the same cell could therefore end up in many other different cells, though often ones which are nearby. It is assumed that neurons are distributed uniformly across the source cell. Therefore, the proportion of neurons which end up in each of the other cells can be calculated. MIIND performs this calculation in two ways, the choice for which is given to the user.

The first method is to use a Monte Carlo approach such that a number of points are randomly placed in the source cell then translated according to the efficacy. A search takes place to find which cells the points were translated to and the proportions are calculated from the number of points in each. For many meshes, a surprisingly small number of points, around 10, is required in each cell to get a good approximation for the transition matrix and the process is therefore quite efficient. As shown in Figure 10, an additional process is required when generating transition matrices using Monte Carlo which includes two further intermediate files, *.fid* and *.lost*. All points must be accounted for when performing the search and in cases where points are translated outside of the mesh, an exhaustive search must be made to find the closest cell. The **lost** command allows the user to speed up this process which is covered in detail in Supplementary Section 6.1 of the Supplementary Material.

The second method translates the actual vertices of each cell according to the efficacy and calculates the exact overlapping area with other cells. The method by which this is achieved is the same as that used to generate the transition matrix of the grid algorithm, described in section 2.1. This method provides much higher accuracy than Monte Carlo but is one order of magnitude slower (it takes a similar amount of time to perform Monte Carlo with 100 points per cell). For some meshes, it is crucial to include very small transitions between cells to properly capture the dynamics which justifies the need for the slower method. It also benefits from requiring no additional user input in contrast to the Monte Carlo method.

In *miind.miindio*, the command **generate-matrix** can be used to automatically generate each *.mat* file. In order to work, there must be a *basename.model* file in the working directory. The **generate-matrix** command takes six parameters which are described in Table 3. Listing 8 shows an example of the **generate-matrix** command. If successful, two files are generated: *basename*.*mat* and *basename*.*lost*.

**Table 3:** Parameters for the **generate-matrix** command in the CLI.

**Parameter name**	**Notes**
*basename*	The shared name of the *.model, .fid* (if required), and generated *.mat* files.
*v_efficacy*	The efficacy value in the *v* (membrane potential) direction. If the parameter *h_efficacy* is used, this should be zero.
*points / precision*	For Monte Carlo, this gives the number of points per cell to use for approximating the transition matrix. For the geometric method, transitions are stored in the *.mat* file to the nearest $\frac{1}{precision}$
*h_efficacy*	The efficacy value in the *h* direction. If the parameter *v_efficacy* is used, this should be zero.
*reset-shift*	The shift in the *h* direction which neurons take when being reset.
*use_geometric*	A boolean flag set to “true” if the geometric method is used and “false” for Monte Carlo.

**Listing 8:**
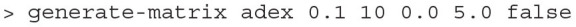
The *miind.miindio* command to generate a matrix using the adex.model file with an efficacy of 0.1 in *v* and a jump of 5.0 in *w* when a neuron spikes. The Monte Carlo method has been chosen with 10 points per cell.

Once **generate-matrix** has completed, a *.mat* file will have been generated and the *.model* file will have been amended to include a *<Reset Mapping>* section. Similar to the reversal mapping in the *.rev* file, the reset mapping describes movement of probability mass from the cells which lie across the threshold potential to cells which lie across the reset potential. If the threshold or reset values are changed but no other change is made to the mesh, it can be helpful to re-run the mapping calculation without having to completely re-calculate the transition matrix. *miind.miindio* provides the command **regenerate-reset** which takes the base name and any new reset shift value (0 if not required) as parameters. This will quickly replace the reset mapping in the *.model* file.

**Listing 9:**
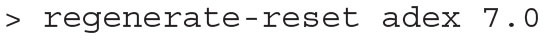
The user may change the *<Threshold>* and *<Reset>* values in the *.model* file (or re-call **generate-model** with different threshold and reset values) then update the existing Reset Mapping. In this case, the adex.model was updated with a reset *w* shift value of 7.0.

With all required files generated, a simulation using the mesh algorithm can now be run in MIIND.

### 3.3. Jump Files

In some models, it is helpful to be able to set the efficacy as a function of the state. For example, to approximate adaptive behaviour where the post synaptic efficacy lowers as the membrane potential increases. Jump files have been used in MIIND to simulate the Tsodyks-Markram (Tsodyks and Markram, 1997) synapse model as described in De Kamps et al. (2019). In the model, one variable/dimension is required to represent the membrane potential, *V*, of the post-synaptic neuron and the second to represent the synaptic contribution, *G*. *G* and *V* are then used to derive the post-synaptic potential caused by an incoming spike. Before generating the transition matrix, each cell can be assigned its own efficacy for which the transitions will be calculated. During generation, Monte Carlo points will be translated according to that value instead of a constant across the entire mesh. When calling the **generate-matrix** command, a separate set of three parameters is required to use this feature. The base name of the model file, the number of Monte Carlo points per cell, and a reference to a *.jump* file which stores the efficacy values for each cell in the mesh.

**Listing 10:**
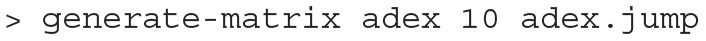
Generate a transition matrix with a jump file in the CLI

As with the files required to build the mesh, the jump file must be user generated as the efficacy values may be non-linear and involve one or both of the dimensions of the model. The format of a jump file is shown in listing 11. The *<Efficacy>* element of the XML file gives an efficacy value for both dimensions of the model and is how the resulting transition matrix will be referenced in the simulation. The *<Translations>* element lists the efficacy in both dimensions for each cell in the mesh.

**Listing 11:**
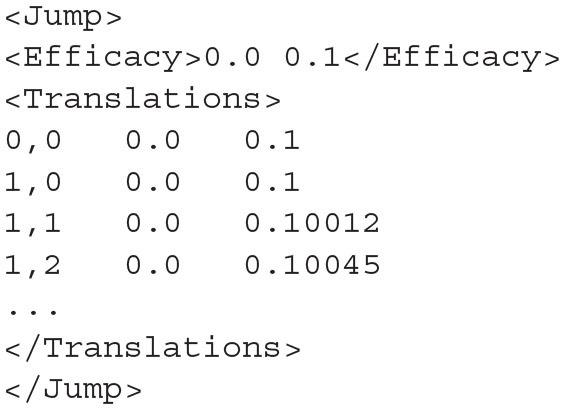
The format of the jump file. Each line in the *<Translations>* block gives the strip,cell coordinates of the cell followed by the *h* efficacy then the *v* efficacy. The *<Efficacy>* element gives a reference efficacy which will be used to reference the transition matrix built with this jump file. It must therefore be unique among jump files used for the same model.

After calling **generate-matrix**, as before, the *.mat* file will be created with the quoted values in the *<Efficacy>* element of the jump file. As with the vanilla Monte Carlo generation, the additional process of tracking lost points must be performed.

## 4. Writing the XML File

MIIND provides an intuitive XML style language to describe a simulation and its parameters. This includes descriptions of populations, neuron models, integration techniques, and connectivity as well as general parameters such as time step and duration. The XML file is split into sections which are sub elements of the XML root node, *<Simulation>*. They are Algorithms, Nodes, Connections, Reporting, and SimulationRunParameter. These elements make up the major components of a MIIND simulation.

### 4.1. Algorithms

An *<Algorithm>* in the XML code describes the simulation method for a population in the network. The nodes of the network represent separate instances of these algorithm elements. Therefore, many nodes can use the same algorithm. Each algorithm has different parameters or supporting files but as a minimum, all algorithms must declare a type and a name. Each algorithm is also implicitly associated with a “weight type.” All algorithms used in a single simulation must be compatible with the weight type as it describes the way that populations interact. The *<WeightType>* element of the XML file can take the values, “double,” “DelayedConnection,” or “CustomConnectionParameters.” Which value the weight type element takes influences which algorithms are available in the simulation and how the connections between populations will be defined. The following sections cover all Algorithm types currently supported in MIIND. Table 4 lists these algorithms and their compatible weight types.

**Table 4:** Compatible weight types for each algorithm type defined in the simulation XML file.

**Algorithm name**	**Double**	**DelayedConnection**	**CustomConnectionParameters**
*RateAlgorithm*	✓	✓	✓
*MeshAlgorithm*		✓	
*MeshAlgorithmCustom*			✓
*GridAlgorithm*			✓
*GridJumpAlgorithm*			✓
*OUAlgorithm*		✓	
*WilsonCowanAlgorithm*	✓		
*RateFunctor*	✓	✓	✓

#### 4.1.1. RateAlgorithm

RateAlgorithm is used to supply a Poisson distributed input (with a given average firing rate) to other nodes in the simulation. It is typically used for simulating external input. The *<rate>* sub-element is used to define the activity value which is usually a firing rate.

**Listing 12:**
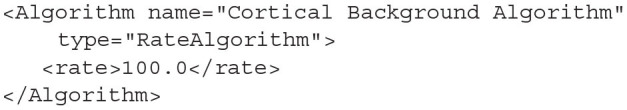
A RateAlgorithm definition with a constant rate of 100 Hz.

#### 4.1.2. MeshAlgorithm and MeshAlgorithmCustom

In section 3.2, we saw how to generate *.model* and *.mat* files. These are required to simulate a population using the mesh algorithm. Algorithm type=MeshAlgorithm tells MIIND to use this technique. The model file is referenced as an attribute to the Algorithm definition. The *TimeStep* child element must match that which was used to generate the mesh. This value is quoted in the model file. As many *MatrixFile* elements can be declared as are required for the simulation, each with an associated .mat file reference.

**Listing 13:**
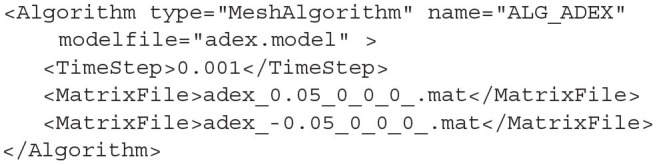
A MeshAlgorithm definition with two matrix files.

MeshAlgorithm provides two further optional attributes in addition to *modelfile*. The first is *tau_refractive* which enables a refractory period and the second is *ratemethod* which takes the value “AvgV” if the activity of the population is to be represented by the average membrane potential. Any other value for *ratemethod* will set the activity to the default average firing rate. The activity value is what will be passed to other populations in the network as well as what will be recorded as the activity for any populations using this algorithm.

When the weight type is set to CustomConnectionParameters, the type of this algorithm definition should be changed to MeshAlgorithmCustom. No other changes to the definition are required.

#### 4.1.3. GridAlgorithm and GridJumpAlgorithm

For populations which use the grid algorithm, the following listing is required. Similar to the MeshAlgorithm, the model file is referenced as an attribute. However, there are no matrix files required as the transition matrix for solving the Poisson master equation is calculated at run time. The transition matrix for the deterministic dynamics, stored in the *.tmat* file, is referenced as an attribute as well. Attributes for *tau_refractive* and *ratemethod* are also available with the same effects as for MeshAlgorithm.

**Listing 14:**
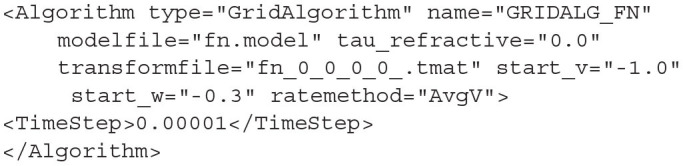
A GridAlgorithm definition using the AvgV (membrane potential) rate method.

GridAlgorithm also provides additional attributes *start_v* and *start_w* which allows the user to set the starting state of all neurons in the population which creates an initial probability mass of 1.0 in the corresponding grid cell at the start of the simulation.

GridJumpAlgorithm provides a similar functionality as MeshAlgorithm when the transition matrix is generated using a jump file. That is, the efficacy applied to each cell when calculating transitions differs from cell to cell. In GridJumpAlgorithm, the efficacy at each cell is multiplied by the distance between the central *v* value of the cell and a user defined “stationary” value. The initial efficacy and the stationary values are defined by the user in the XML *<Connection>* elements. GridJumpAlgorithm is useful for approximating populations of neurons with a voltage dependent synapse.

**Listing 15:**
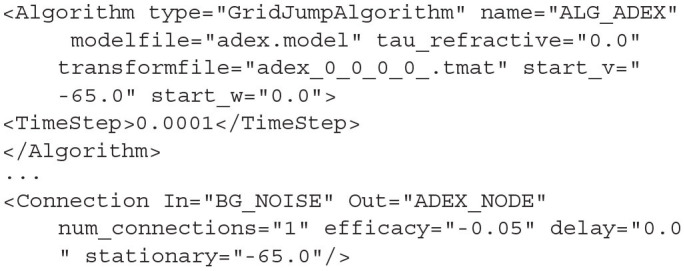
A GridJumpAlgorithm definition and corresponding Connection with a “stationary” attribute. The efficacy at each grid cell will equal the original efficacy value (−0.05) multiplied by the difference between each cell's central v value and the given stationary value (−65)

#### 4.1.4. Additional Algorithms

MIIND also provides OUAlgorithm and WilsonCowanAlgorithm. The OUAlgorithm generates an Ornstein–Uhlenbeck process (Uhlenbeck and Ornstein, 1930) for simulating a population of LIF neurons. The WilsonCowanAlgorithm implements the Wilson-Cowan model for simulating population activity (Wilson and Cowan, 1972). Examples of these algorithms are provided in the examples directory of the MIIND repository (*examples/twopop* and *examples/model_archive/WilsonCowan*).

One final algorithm, RateFunctor, behaves similarly to RateAlgorithm. However, instead of a rate value, the child value defines the activity using a C++ expression in terms of variable, *t*, representing the simulation time.

**Listing 16:**
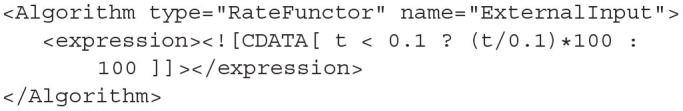
A RateFunctor algorithm definition in which the firing rate linearly increases to 100 Hz over 0.1 s and remains at 100 Hz thereafter.

A CDATA expression is not permitted when using MIIND in Python or when calling *miind.run*. However, RateFunctor can still be used with a constant expression (although this has no benefit beyond what RateAlgorithm already provides). CDATA should only be used when MIIND is built from source (not installed using pip) and the MIIND API is used to generate C++ code from an XML file.

### 4.2. Nodes

The *<Node>* block lists instances of the Algorithms defined above. Each node represents a single population in the network. To create a node, the user must provide the name of one of the algorithms defined in the algorithm block which will be instantiated. A name must also be given to uniquely identify this node. The type describes the population as wholey inhibitory, excitatory, or neutral. The type dictates the sign of the post synaptic efficacy caused by spikes from this population. Setting the type to neutral allows the population to produce both excitatory and inhibitory (positive and negative) synaptic efficacies. For most algorithms, the valid types for a node are *EXCITATORY, INHIBITORY*, and *NEUTRAL*. *EXCITATORY_DIRECT* and *INHIBITORY_DIRECT* are also available but mean the same as *EXCITATORY* and *INHIBITORY*, respectively.

**Listing 17:**
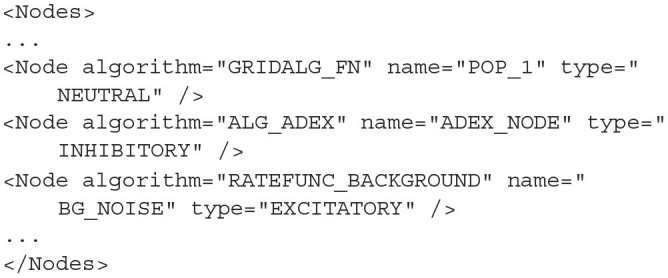
Three nodes defined in the Nodes section using the types *NEUTRAL, INHIBITORY*, and *EXCITATORY*, respectively.

Many nodes can reference the same algorithm to use the same population model but they will behave independently based on their individual inputs.

### 4.3. Connections

The connections between the nodes are defined in the *<Connections>* sub-element. Each connection can be thought of as a conduit which passes the output activity from the “In” population node to the “Out” population node. The format used to define the connections is dependent on the choice of *WeightType*. When the type is *double*, connections require a single value which represents the connection weight. This will be multiplied by the output activity of the In population and passed to the Out population. The sign of the weight must match the In node's type definition (*EXCITATORY, INHIBITORY, NEUTRAL*).

**Listing 18:**
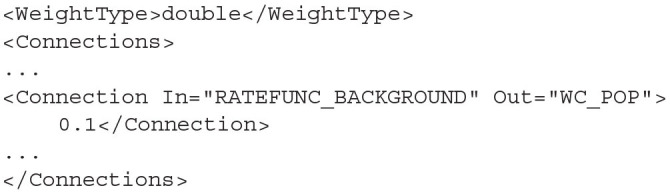
A simple double WeightType Connection with a single rate multiplier.

Many algorithms use the *DelayedConnection* weight type which requires three values to define each connection. The first is the number of incoming connections each neuron in the Out population receives from the In population. This number is effectively a weight and is multiplied by the output activity of the In population. For example, if the output firing rate of an In population is 10 Hz and the number of incoming connections is set to 10, the effective average incoming spike rate to each neuron in the Out population will be 100 Hz. The second value is the post synaptic efficacy whose sign must match the type of the In population. If the Out population is an instance of MeshAlgorithm, the efficacy must also match one of the provided *.mat* files. The third value is the connection delay in seconds. The delay is implemented in the same way as the refractory period in the mesh and grid algorithms. The output activity of the In population is placed at the beginning of the queue and shifted toward the end of the queue over subsequent iterations. The input to the Out population is taken as the linear interpolation between the final two values in the queue.

**Listing 19:**
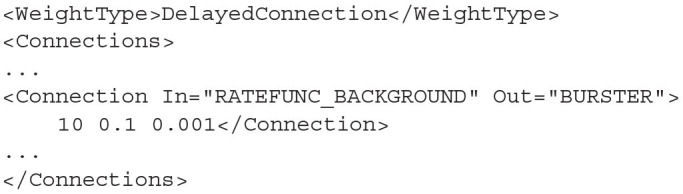
A DelayedConnection with number of connections = 10, efficacy = 0.1, and delay of 1ms.

With the addition of GridAlgorithm, there was a need for a more flexible connection type which would allow custom parameters to be applied to each connection. When using the *CustomConnectionParameters* weight type, the key-value attributes of the connections are passed as strings to the C++ implementation. By default, custom connections require the same three values as *DelayedConnection*: *num_connections, efficacy*, and *delay*. *CustomConnectionParameters* can therefore be used with mesh algorithm nodes as well as grid algorithm nodes although MeshAlgorithm definitions must have the type attribute set to MeshAlgorithmCustom instead.

**Listing 20:**
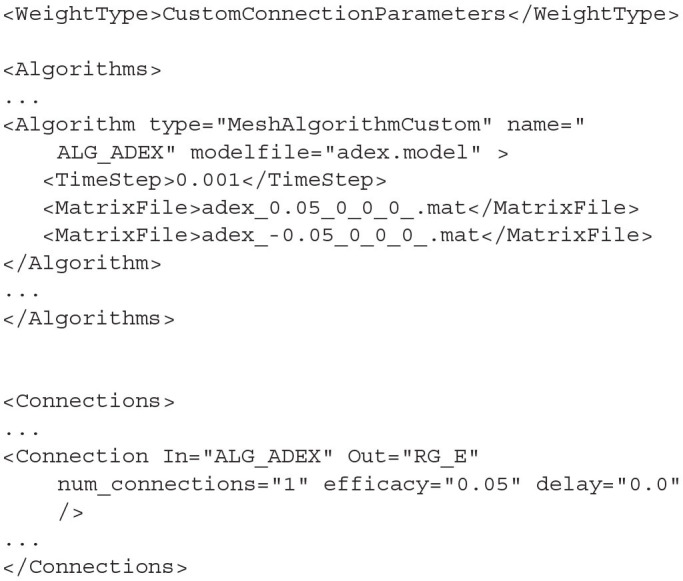
A MeshAlgorithmCustom definition for use with WeightType=CustomConnectionParameters and a Connection using the num_connections, efficacy, and delay attributes.

Other combinations of attributes for connections using CustomConnectionParameters are available for use with specific specialisations of the grid algorithm which are discussed in Supplementary Section 4 of the Supplementary Material. Any number of attributes are permitted but they will only be used if there is an algorithm specialisation implemented in the MIIND code base.

### 4.4. SimulationRunParameter

The *<SimulationRunParameter>* block contains parameter settings for the simulation as a whole. The sub-elements listed in Table 5 are required for a full definition. Although most of the sub-elements are self explanatory, *t_step* has the limitation that it must match or be an integer multiple of all time steps defined by any MeshAlgorithm and GridAlgorithm instances. *master_steps* is used only for the GPGPU implementation of MIIND (section 5). It allows the user to set the number of Euler iterations per time step to solve the master equation. By default, the value is 10. However, to improve accuracy or to avoid blow-up in the case where the time step is too large or the local dynamics are unstable, *master_steps* should be increased.

**Table 5:** The required sub-elements for the *SimulationRunParameter* section of the XML simulation file.

**Element**	**Notes**
*SimulationName*	The name of the simulation.
*t_end*	The simulation end time.
*t_step*	The time step of the simulation.
*name_log*	A file name for logging. The file is stored in the output directory of the simulation.
*master_steps*	The number of Euler iterations per time step used to solve the mater equation in the GPGPU implementation.

### 4.5. Reporting

The *<Reporting>* block is used to describe how output is displayed and recorded from the simulation. There are three ways to record output from the simulation: Density, Rate, and Display. The *<Rate>* element takes the node *name* and *t_interval* as attributes and creates a single file in the output directory. *t_interval* must be greater than or equal to the simulation time step. At each *t_interval* of the simulation, the output activity of the population is recorded on a new line of the generated file. Although the element is called “Rate,” if average membrane potential has been chosen as the activity of this population, this is what will be recorded here. *<Density>* is used to record the full probability density of the given population node. As density is only relevant for the population density technique, it can only be recorded from nodes which instantiate the mesh or grid algorithm types. The attributes are the node *name, t_start, t_end*, and *t_interval* which define the simulation times to start and end recording the density at the given interval. A file which holds the probability mass values for each cell in the mesh or grid will be created in the output directory for each *t_interval* between *t_start* and *t_end*. Finally, the *<Display>* element can be used to observe the evolution of the probability density function as the simulation is running. If a *Display* element is added in the XML file for a specific node, when the simulation is run, a graphical window will open and display the probability density for each time step. Again, display is only applicable to algorithms involving densities. Enabling the display can significantly slow the simulation down. However, it is useful for debugging the simulation and furthermore, each displayed frame is stored in the output directory so that a movie can be made of the node's behaviour. How to generate this movie is discussed later in section 7.1.

**Listing 21:**
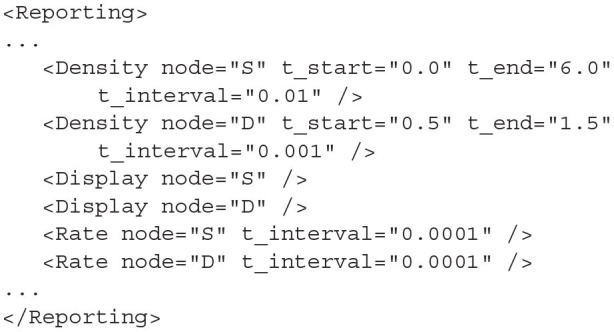
A set of reporting definitions to record the probability densities and rates of two populations, S and D. The densities will also be displayed during simulation.

### 4.6. Variables

The *<Simulation>* element can contain multiple *<Variable>* sub-elements each with a unique name and value. Variables are provided for the convenience of the user and can replace any values in the XML file. For example, a variable named *TIME_END* can be defined to replace the value in the *t_end* element of the *SimulationRunParameter* block. When the simulation is run, the value of *t_end* will be replaced with the default value provided in the Variable definition. Using variables makes it easy to perform parameter sweeps where the same simulation is run multiple times and only the variable's value is changed. How parameter sweeps are performed is covered in the Supplementary Section 8. All values in a MIIND XML script can be set with a variable name. The type of the Variable is implicit and an error will be thrown if, say, a non-numerical value is passed to the tau_refractive attribute of a MeshAlgorithm object.

**Listing 22:**
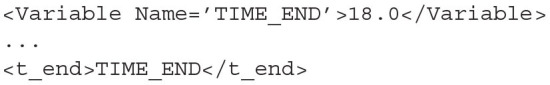
A Variable definition. TIME_END has a default value of 18.0 and is used in the *t_end* parameter definition.

## 5. MIIND on the GPU

The population density techniques of the mesh and grid algorithms rely on multiple applications of the transition matrix which can be performed on each cell in parallel. This makes the algorithms prime candidates for parallelisation on the graphics card. In the CPU versions, the probability mass is stored in separate arrays, one for each population/node in the simulation. For the GPGPU version, these are concatenated into one large probability mass vector so all cells in all populations can be processed in parallel. From the user's perspective, switching between CPU and GPU implementations is trivial. In the XML file for a simulation which uses MeshAlgorithm or GridAlgoirithm, to switch to the vectorised GPU version, the Algorithm types must be changed to MeshAlgorithmGroup and GridAlgorithmGroup. All other attributes remain the same. Only MeshAlgorithmGroup, GridAlgorithmGroup, and RateFunctor/RateAlgorithm types can be used for a vectorised simulation. When running a MIIND simulation containing a group algorithm from a Python script, instead of importing *miind.miindsim, miind.miindsimv* should be used. The Python module *miind.run* is agnostic to the use of group algorithms so can be used as shown previously.

**Listing 23:**
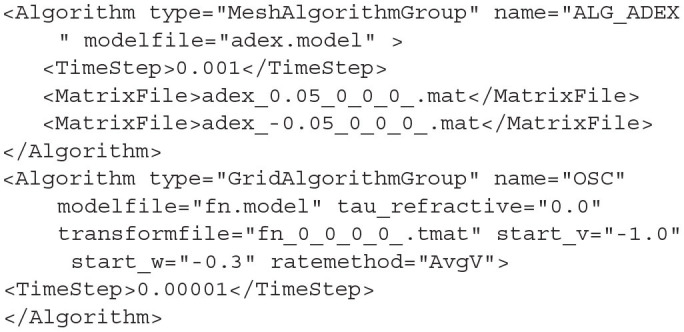
A MeshAlgorithmGroup definition is identical to a MeshAlgorithm definition except for the type.

The GPGPU implementation uses the Euler method to solve the master process during each iteration. It is, therefore, susceptible to blow-up if the time step is large or if the local dynamics of the model are stiff. The user has the option to set the number of euler steps taken each iteration using the *master_steps* value of the SimulationRunParameter block in the XML file. A higher value reduces the likelihood of blow-up but increases the simulation time.

In order to run the vectorised simulations, MIIND must be running on a CUDA enabled machine and have CUDA enabled in the installation (CUDA is supported in the Windows and Linux python installations). Supplementary Section 3 in the Supplementary Material goes into greater detail about the systems architecture differences between the CPU and GPU versions of the MIIND code. Using the “Group” algorithms is recommended if possible as it provides a significant performance increase. As shown in De Kamps et al. (2019), with the use of the GPGPU, a population of conductance based neurons in MIIND performs comparably to a NEST simulation of 10,000 individual neurons but using an order of magnitude less memory. This allows MIIND to simulate many thousands of populations on a single PC.

## 6. Running a MIIND Simulation in Python

As demonstrated in the quick start guide, the command **python -m miind.run** takes a simulation XML file as a parameter and runs the simulation. A similar script may be written by the user to give more control over what happens during a simulation and how output activity is recorded and processed. It even allows MIIND simulations to be integrated into other Python applications such as TVB (Sanz Leon et al., 2013) so the population density technique can be used to solve the behaviour of nodes in a brain-scale network (see section 9.2). To run a MIIND simulation in a Python script, the module *miind.miindsim* must be imported (or *miind.miindsimv* if the simulation uses MeshAlgorithmGroup or GridAlgorithmGroup and therefore requires CUDA support). Listing 24 shows an example script which uses the following available functions to control the simulation.

**Listing 24:**
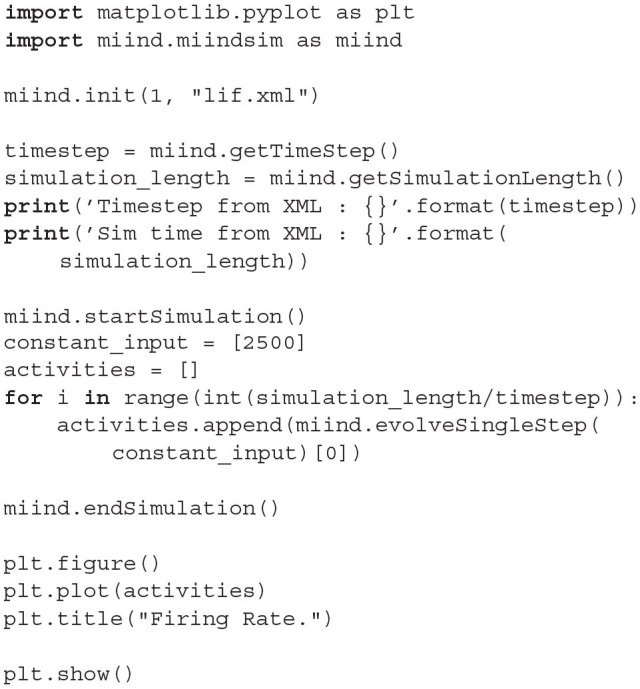
A simple python script for running a MIIND simulation and plotting the results.

### 6.1. init(node_count,simulation_xml_file,…)

The *init* function should be called first once the MIIND library has been imported. This sets up the simulation ready to be started. The *node_count* parameter allows for multiple instantiations of the simulation to be run simultaneously. The Nodes, Connections, and Reporting blocks from the simulation file will be duplicated, effectively running the same model *node_count* times simultaneously in the same simulation. This functionality was included to allow TVB to run the simulation defined in the XML file multiple times (see section 9.2). The *simulation_xml_file* parameter gives the name of the simulation xml file to be run. If the file has any variables defined, these are made available in Python as additional parameters to the *init* function. In this way, the use of XML variables can be used for parameter sweeps. All variables must be passed as strings. If a variable is not set in the call to *init*, the default value defined in the XML file will be used.

**Listing 25:**
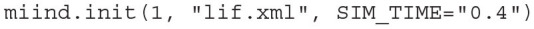
Calling init for a MIIND simulation lif.xml with the Variable SIM_TIME set to 0.4.

### 6.2. getTimeStep() and getSimulationLength()

Once *init* has been called, the functions *getTimeStep* and *getSimulationLength* can be used to extract the time step and simulation length in seconds from the simulation, respectively. The Python script controls when each iteration of the MIIND simulation is called and so it needs to know the total number of iterations to make. Furthermore, it can be useful for integration with other systems to know these values.

### 6.3. startSimulation()

*startSimulation* indicates in the Python script that the simulation should be initialised ready for the simulation loop to be called.

### 6.4. evolveSingleStep(input)

By calling *evolveSingleStep* in the Python script, the MIIND simulation will move forward one time step. This function takes a list of numbers as a parameter. The list corresponds to inputs to the population nodes in the MIIND simulation. In this way, the user may control the behaviour of the simulation from the Python script during the simulation. The *evolveSingleStep* function also returns a list of numbers which are the output activities of the population nodes. Section 6.6 provides more information about how to use the input and output of this function. *evolveSingleStep* should be called in a loop which will run the same number of iterations as would be expected if the XML file were run in MIIND directly, that is, the simulation length divided by the time step.

### 6.5. endSimulation()

It is good practice to call *endSimulation* once all iterations of the simulation have been performed. This allows MIIND to clean up and to print the performance statistics to the console.

### 6.6. Additional XML Code for Python Support

Although it is still possible to use *RateFunctor* or *RateAlgorithm* to set input rates to populations in a Python MIIND simulation, *evolveSingleStep()* provides a means to pass the input rates as a parameter so that more complex input patterns can be used. In order to indicate that a population will receive input externally from the Python script [via the list input to *evolveSingleStep()*] a special connection type must be defined in the *<Connections>* section of the XML.

**Listing 26:**
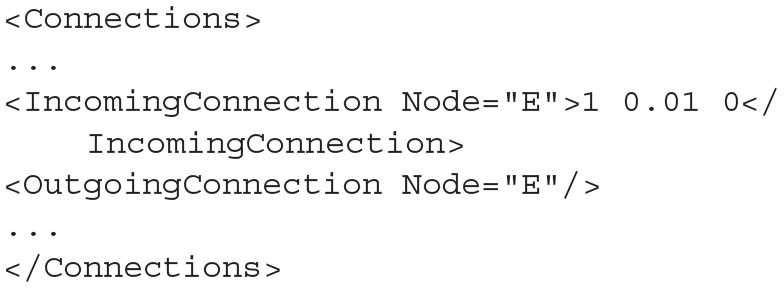
Special connection types for use in Python.

Listing 26 defines an input to node E which will be interpreted as a DelayedConnection with the number of connections equal to 1 and a post synaptic efficacy of 0.01. No delay is defined here although it is permitted. OutgoingConnections are used to declare which nodes in the population network will pass their activity back to the Python script after each iteration. If the two connections in the listing are the only instances of IncomingConnection and OutgoingConnection, then the *evolveSingleStep* function will expect as a parameter, a list with one numeric value to represent the incoming rate to node E. *evolveSingleStep* will return a list with a single numeric value representing the activity of node E. In cases where there are more than one IncomingConnection, the order of values in the Python list parameter to *evolveSingleStep* is the same as the order of IncomingConnections defined in the XML. Similarly with OutgoingConnections, the order of the list of activities returned from *evolveSingleStep* is the same as the order of declaration in the XML file.

## 7. Using the CLI to Quickly View Results

Once a simulation has been run, either using *miind.run* or from a user written Python script, the *miind.miindio* CLI can be used to quickly plot the recorded results. As mentioned, the commands used in *miindio* are based on the module *miind.miind_api* and are reproducible in a Python script. However, it can be convenient to be able to run them directly from the command line to aid fast prototyping and bug fixing of models and simulations. The following section lists some common commands in the CLI and their usage. The accompanying files for this example are in the *examples/cli_plots* directory. The following command starts the CLI and presets the user with a prompt:

**Listing 27:**
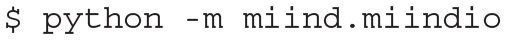
Run the CLI.

When *miind.miindio* is called for the first time in a working directory, the user must identify the XML file which will describe the current working simulation. MIIND stores a reference to this file in a settings file in the working directory so that all subsequent commands will reference this simulation. Even if *miind.miindio* is quit and restarted, the current working simulation will be used as the context for commands until a new current working simulation is defined or if it is called in a different directory. The user can set the current working simulation with the **sim** command.

**Listing 28:**
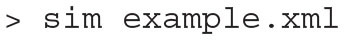
Load a simulation file in the CLI.

Calling **sim** without a parameter will list information about the current working simulation such as the output directory, XML file name and provide a list of the defined variables and nodes.

During the simulation, MIIND generates output files according to the requirements of the *<Recording>* object of the XML file which could include the average firing rate of population nodes or their densities at each time interval. The average firing rate can be plotted from the CLI using the **rate** command followed by the name of the population node. To be reminded of the node names, the user can call **sim** or **rate** without parameters.

**Listing 29:**
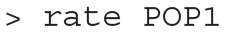
Plot the rate of population POP1 in the CLI.

**Figure 11:**
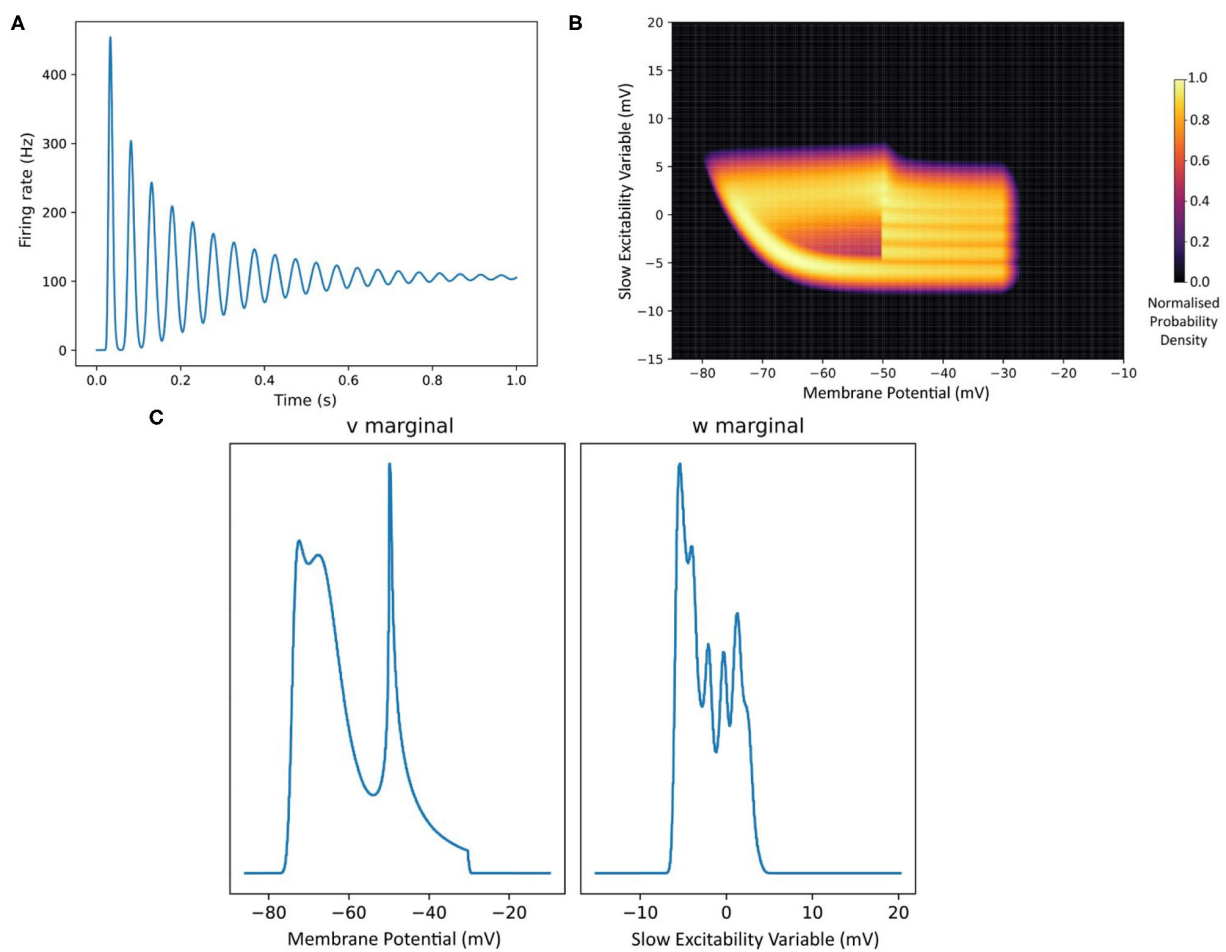
**(A)** The average firing rate of a population produced by calling the **rate** command. **(B)** A density plot (normalised to the maximum density value) of the population produced by calling the **plot-density** command. **(C)** The marginal density plots produced by calling the **plot-marginals** command.

Even while a simulation is running, calling **rate** in the CLI will plot the recorded activity up to the latest simulated time point. This is useful to keep an eye on the simulation as it progresses without waiting for completion. An example of the plots produced by **rate** is shown in Figure 11A.

For populations using the grid or mesh algorithms, the user can call the **plot-density** command with parameters identifying the required node name and simulation time.

**Listing 30:**
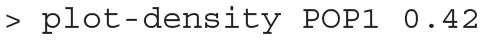
Plot the probability density of population POP1 at time 0.42 s in the CLI.

This command renders the mesh or grid and its population density at the given simulation time. When reading the simulation time parameter in the command, MIIND expects the time to be an integer multiple of the time step and to be expressed up to its least significant figure (for example, 0.1 instead of 0.10). Again, this command can be run during a simulation providing the time has been simulated. An example of a density plot is shown in Figure 11B.

Similar to **plot-density**, **plot-marginals** can be used to display the marginal densities of a given population at a given time. Both marginals are plotted next to each other. The details of how marginal densities are calculated are explained in the Supplementary Section 5. Figure 11C shows an example of a marginal density plot.

**Listing 31:**
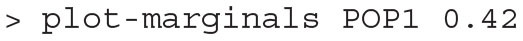
Plot the marginal distributions of population POP1 at time 0.42s in the CLI.

### 7.1. Generate a Density Movie

If, in the XML file *<Recording>* section, the *<Display>* element is added for a given population, the output directory will be populated with still images of density plots at each time step. Once the simulation is complete, calling **generate-density-movie** in the CLI will produce an MP4 movie file made from the still images. The parameters are the node name followed by the size of the square video frame in pixels. The third parameter is the desired time to display each image (every time step of the simulation) in seconds. If the video should be the same length as the simulation time, then this parameter should match the time step of the simulation. By changing the value, the video time can be altered. For example, if the parameter is set to 0.01 for a simulation with time step 0.001, then the video length will be 10 times the length of the simulation. Finally, a name for the video file must be given.

**Listing 32:**
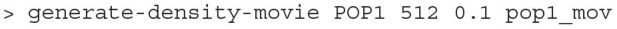
Generate a movie from the display images of population POP1 with a size of 512 pixels at a simulation replay time step of 0.1 s.

The movie file will be created in the working directory of the simulation. A movie of the marginal density plots can also be created using the **generate-marginal-movie** command which takes the same parameters. As each marginal plot must be generated from the density output, this takes a considerably longer time than for the density movie.

**Listing 33:**
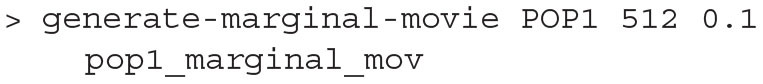
Generate a marginals movie from the density files of population POP1 with a size of 512 pixels at a simulation replay time step of 0.1 s.

## 8. Description of MIIND's Architecture and Functionality

**Figure 12:**
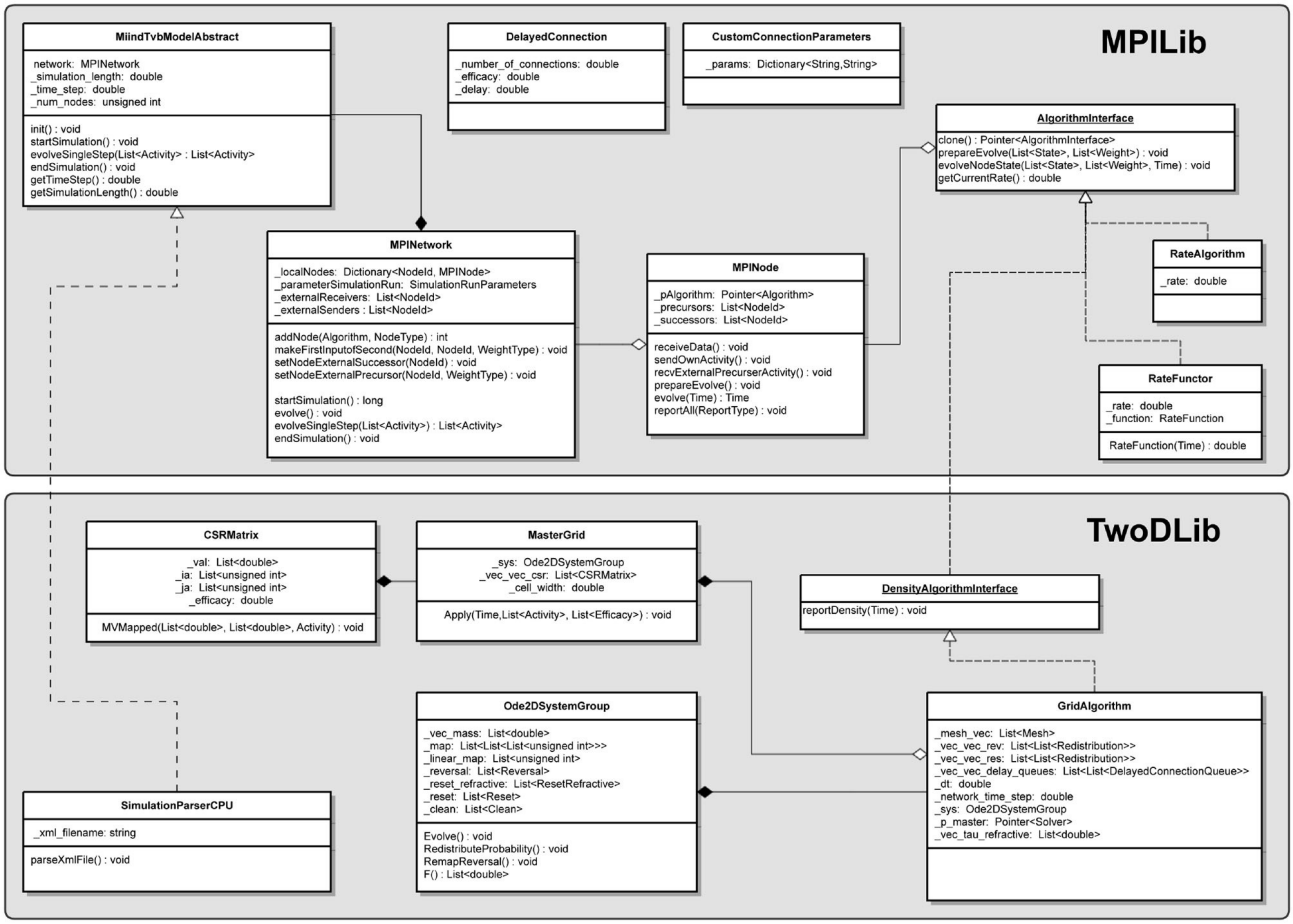
A minimal UML diagram of MIIND. The two major libraries, MPILib and TwoDLib, are represented.

The main architectural concerns in MIIND relate to the two C++ libraries, MPILib and TwoDLib. MPILib is responsible for instantiating and running the simulation. TwoDLib contains the CPU implementations of the grid and mesh algorithms. It is also responsible for generating transition matrices. Of the remaining libraries, GeomLib contains a population density technique implementation of neuron models with one time dependent variable, although it is also possible and indeed preferable to use the TwoDLib code for one dimensional models. EPFLLib and NumtoolsLib contain helper classes and type definitions. Figure 12 shows a reduced UML diagram of the MIIND C++ architecture. The aim of this section is to give a brief overview of the C++ MIIND code as a starting point for developers. The CUDA implementation of MIIND is similar in structure to the CPU solution and is available in the CudaTwoDLib and MiindLib libraries. A description of the differences is given in Supplementary Section 3 of the Supplementary Material.

### 8.1. MPILib

The MPINetwork class in MPILib represents a simulation as a whole and is instantiated in the *init* function of the SimulationParserCPU class which is a specialisation of MiindTvbModelAbstract. *init* is called from the Python module and, as the name suggests, MiindTvbModelAbstract was originally written with the aim of Python integration into TVB. MPINetwork exposes member functions for building a network of nodes where each node is an instance of a neuron population which can be connected together so that the output activity from one population is input to another. The class also contains all of the simulation parameters such as the simulation length and time step. Finally, the MPINetwork class exposes a function to run the simulation in its entirety or take a single evolve step for use in an external control loop.

Each node in the population network is represented by an instance of the MPINode class. A node has a name and an ID which is used to uniquely identify it in the simulation. A node also contains an implementation of AlgorithmInterface performing the integration technique required for this population (for example, GridAlgorithm or MeshAlgorithm). The *NodeType* describes whether a population should be thought of as excitatory or inhibitory. As discussed earlier, MIIND performs a validation check that the synaptic efficacy from a node is positive or negative, respectively (or neutral). During each iteration, each node is responsible for consolidating the activity of all input connections, calling the integration step in the AlgorithmInterface implementation, and reporting the density and output activity (the average firing rate or membrane potential).

In MPILib, a number of implementations of AlgorithmInterface are defined which can be instantiated in a node. Implementations of AlgorithmInterface are responsible for the lion's share of the computation in MIIND as this is where the integration of the model is performed. The interface is extremely simple, providing a function to set parameters, an optional function for a preamble before each iteration, and the *evolveNodeState* function to be called every time step. GridAlgorithm and MeshAlgorithm are implementations of this interface defined in TwoDLib. MPILib and GeomLib hold the implementations of the remaining algorithms available to the user which were discussed in section 4. Finally, the weight types, DelayedConnection and CustomConnectionParameters are also defined in MPILib. All classes are C++ templates which take the weight type as a parameter to avoid code duplication and to enforce that only algorithms with the same weight type can be used together.

### 8.2. TwoDLib

As with the population models in MPILib and GeomLib, GridAlgorithm and MeshAlgorithm are implementations of the AlgorithmInterface. We will focus here on the grid algorithm implementation although the mesh algorithm uses the same structures or specialisations of those structures to perform similar tasks as set out in section 3. GridAlgorithm is supported by two important classes. **Ode2DSystem** transfers probability mass according to the reset mapping of the *.model* file and calculates the average firing rate of the population. In MeshAlgorithm, Ode2DSystem also performs the pointer update for shifting probability mass down the strips of the mesh. **MasterGrid** is responsible for solving the Poisson master equation using a transition matrix calculated at simulation time based on the desired efficacy and grid cell size. For each iteration, the function *evolveNodeState* is called which performs the main steps of the population density algorithm.

First, in GridAlgorithm, the deterministic dynamics are solved by applying the pre-generated transition matrix once. The second step is a call to *Ode2DSystem.RedistributeProbability()* to perform any reset mappings for probability mass which appeared in the threshold cells last iteration. This step is useful for neuron models, such as leaky integrate and fire, which contain an instruction to reset one or more variables to a different value upon reaching a threshold.

The third step calls on the MasterGrid class to solve the master equation for the incoming Poisson spike rates from every incident node. MasterGrid begins with the current state of the probability mass distribution across the grid, that is, the probability mass values of each cell in the grid. As described in section 2, every cell has the same relative transition of probability mass due to a single incoming spike. For the whole grid, this single transition is duplicated into a transition matrix which can be applied to the full probability mass vector. Because there are at most two cells into which probability mass is transferred, this matrix is extremely sparse and can be stored efficiently in a compressed sparse row (CSR) matrix. In the mesh algorithm, this matrix is loaded from the *.mat* file.

MeshAlgorithm requires a fourth step to transfer probability mass from the ends of strips to stationary cells subject to a reversal mapping generated during the pre-processing phase. This is discussed in the Supplementary Section 6.

Finally, SimulationParserCPU is an extension of the MiindTvbModelAbstract class used to parse the simulation XML file and instantiate an MPINetwork object with the appropriate nodes and connections. Its extensions of the functions declared in MiindTvbModelAbstract are exposed to the Python module to be called from a Python script.

## 9. Discussion

### 9.1. MIIND Fulfills a Need for Insight Into Neural Behaviour at Mesoscopic Scales

The MIIND population density technique allows researchers to simulate population level behaviour by defining the behaviour of the underlying neurons. This is in contrast to many rate based models which describe the population behaviour directly. An example of how population behaviour can differ from the underlying neuron model can be seen in the behaviour of a population of bursting neurons such as the Izhikevich simple model. A single Izhikevich neuron with a constant input current or input spike rate oscillates between a bursting period of repeated firing and a quiescent period of no firing. The average behaviour of a population of Izhikevich neurons is different. Initially, all neurons are synchronised, they burst and quiesce at the same time producing an oscillatory pattern of average firing rate in the population. However, due to the random nature of Poisson input spikes, the neurons de-synchronise over time and the average firing rate of the whole population damps to a constant value because only a subset of neurons are bursting at any one time. Figure 11A shows the damping of the output firing rate oscillations and the “desynchronised” density of a population of Izhikevich simple neurons.

### 9.2. TVB Integration

The Virtual Brain (Sanz Leon et al., 2013) and MIIND are both systems which facilitate the development of neural mass or mean field population models with explicit descriptions of how multiple populations are connected. Using these systems, the complex dynamics arising from the interaction of populations can be studied. TVB provides a framework to describe a network of nodes (the connectivity) which, while it can be abstract, generally represents regions of the human or primate brain. Connections between nodes represent white matter tracts which transfer signals from one node to the next based on length and propagation speed. TVB also allows the description of “coupling” functions which modulate these signals as they pass from one node to another. Typically, the number of nodes is in the order of 100 or so. However, TVB also allows for the definition of a “surface” which can be associated with 10s of thousands of nodes to simulate output from common medical recording techniques such as EEG and BOLD fMRI. TVB has impressive clinical relevance as well as supporting more theoretical neuroscience research. Users can build simulations using the graphical user interface or directly using the Python source code.

While MIIND and TVB have many functional similarities, both have differing strengths with respect to the underlying simulation techniques and surrounding infrastructure. It was therefore clear that integrating the smaller system, MIIND, into the more developed infrastructure of TVB might yield benefits from both.

Although it is possible to model delayed connections and synaptic dynamics between populations in MIIND, TVB provides a comprehensive method of defining such structures and behaviours through the connectivity network and coupling functions. Some users of MIIND may find it useful and appropriate to house their simulations in such a structure.

**Figure 13:**
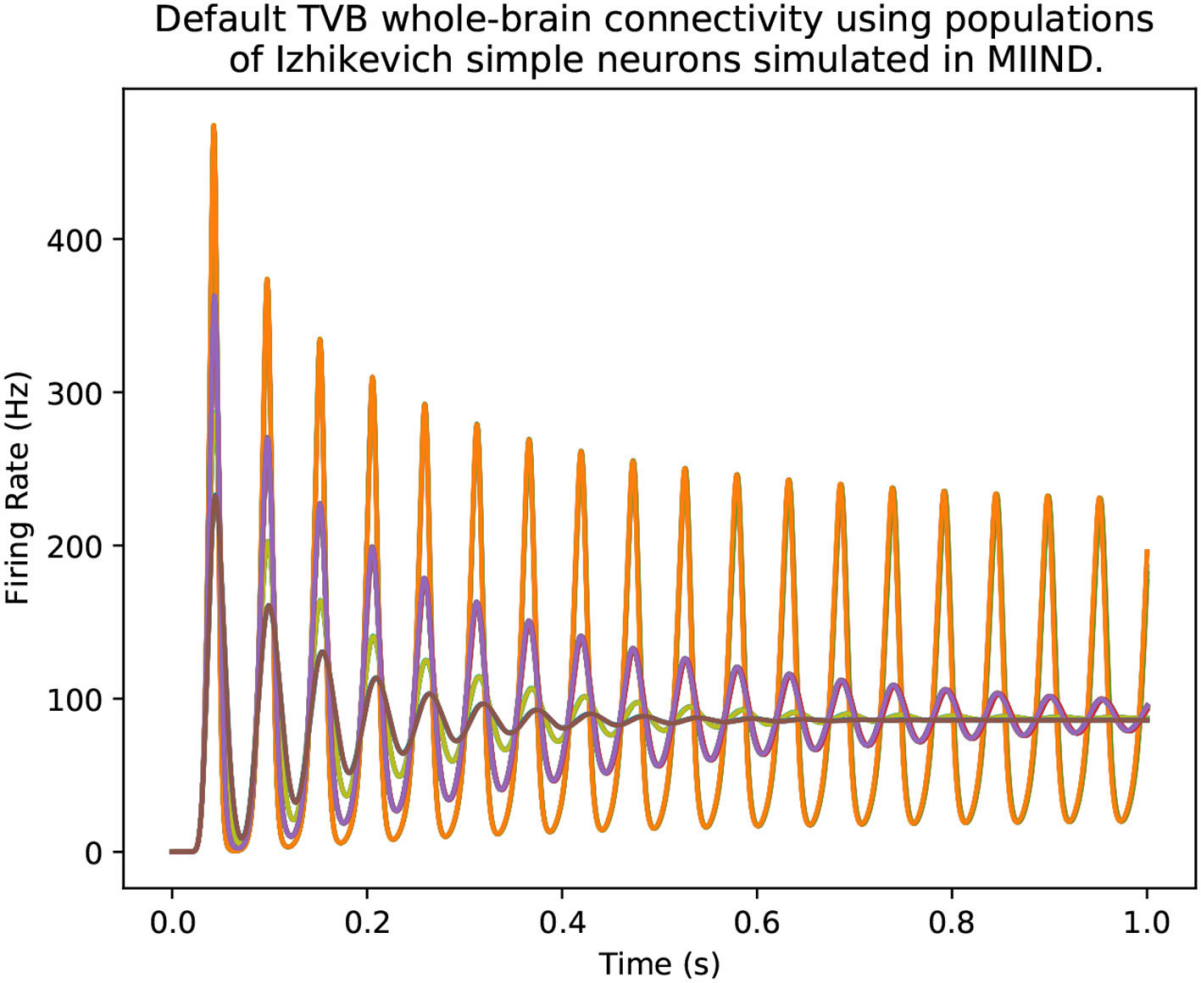
The firing rates of 76 nodes from the default TVB connectivity simulation. Each node is a population of Izhikevich simple neurons simulated using MIIND. The majority of nodes produce oscillations which decay to a constant average firing rate. However, a subset of nodes remain in an oscillating state.

TVB uses a number of model classes to describe the behaviour of the nodes in a network. When the simulation is run, an instantiation of a specified model class takes the signals which have passed through the network to arrive at each node and integrates forward by one time step (depending on the integration method). In order to use MIIND nodes in TVB, a specialised model class was created to import the MIIND Python library, instantiate it, then make a call to *evolveSingleStep()* in place of the integration function. The inputs and outputs of *evolveSingleStep()* are treated by TVB as any other model. As the MIIND Python library takes a simulation file name as a parameter to its *init* function, a single additional model class is all that is required to expose any MIIND simulation to TVB. Figure 13 shows the results from a simulation of the TVB default whole-brain connectivity with populations of Izhikevich simple neurons in MIIND. The script and simulation files are available in the *examples/miind_tvb* directory of the MIIND repository. Both TVB and MIIND must be installed to successfully run the example.

### 9.3. Reasoning About Probability Density Instead of Populations of Individual Neurons Simplifies Output Analysis

The output firing rate or membrane potential of a MIIND population which uses the mesh algorithm or grid algorithm is devoid of any variation which you would see from a population of individual neurons. This is because the effect of Poisson generated input spike trains is applied to a probability density function, effectively an infinite population of neurons. Spike train inputs to a finite population of neurons produces variation in how individual neurons move through state space resulting in noisy output rates at the population level. While this can be mitigated using a larger number of neurons, the use of smoothing techniques, or curve fitting, MIIND requires none of these methods to produce an output which is immediately clear to interpret. For example, MIIND was used to build and simulate a spinal circuit model using populations of integrate and fire neurons (York et al., 2021). The average firing rates of the populations were used to compare patterns of activity with results from an EMG experiment. As the patterns to be observed were on the order of seconds, there was no need to capture faster variation in activity from the simulation and indeed, a direct simulation would have produced output which may have obscured these patterns.

MIIND has also been used to simulate central pattern generator models which rely on mutually inhibiting populations of bursting neurons. The interaction of the two populations significantly influences their sub-threshold dynamics. In particular, it can be difficult to identify the dynamics responsible for the swapping of states from bursting to quiescent (escape or release). Observing the changing probability density function during the simulation makes it very clear how the two populations are behaving.

### 9.4. Handling Noise

A major benefit of MIIND's population density technique is the ability to observe the effect of noise on a population, and to manipulate noise in an intuitive way. For a given simulation, the Poisson distributed input to a population causes a spread of probability mass across the state space as some neurons receive many spikes, and some receive fewer. It is explained in de Kamps (2013) how the Poisson input causes a mean increase in membrane potential equal to the product of the post synaptic efficacy, *h*, and the average input rate, ν. It causes a variance equal to ν*h*^2^. *h* and ν can therefore be set such that the mean remains the same but the variance changes to observe the effect of noise on the population.

Another simple way to increase the variance of the population is to introduce two additional inputs with equal rates and opposite post-synaptic efficacies. Again, the mean increase caused by the input remains unchanged but the variance can be increased significantly and this requires only a small change to the XML simulation file.

### 9.5. A Model Agnostic System at the Population Level Makes Prototyping Quick and Intuitive

Because MIIND provides insight of how a neuron model produces behaviour at the population level, it is beneficial that the grid algorithm enables the user to quickly reproduce the *.model* and *.tmat* files if the underlying neuron model needs to be changed. An example of this can be observed in a half-centre oscillator made of a pair of mutually inhibiting populations of bursting neurons. The frequency of oscillation can be made dependent or independent of the input spike rate by including a limit on the slow excitability variable of the underlying neuron model. To make this change, the user can alter the neuron model then rebuild the *.model* and *.tmat* file and no change to the population level network is required.

### 9.6. DiPDE

DiPDE (Iyer et al., 2013; DiPDE, 2015) is an alternative implementation of the population density technique for one dimensional neuron models. It does not employ the “mesh” discretisation method used in the MIIND mesh algorithm and has primarily been used with populations of leaky integrate and fire neurons. DiPDE can be used to simulate the Potjans-Diesmann microcircuit model (Cain et al., 2016) which shows good agreement with MIIND (Figure 5). MIIND is a much larger application than DiPDE because it allows users to design their own underlying neuron models for each population using either the mesh or grid algorithms.

### 9.7. Future Work

**Figure 14:**
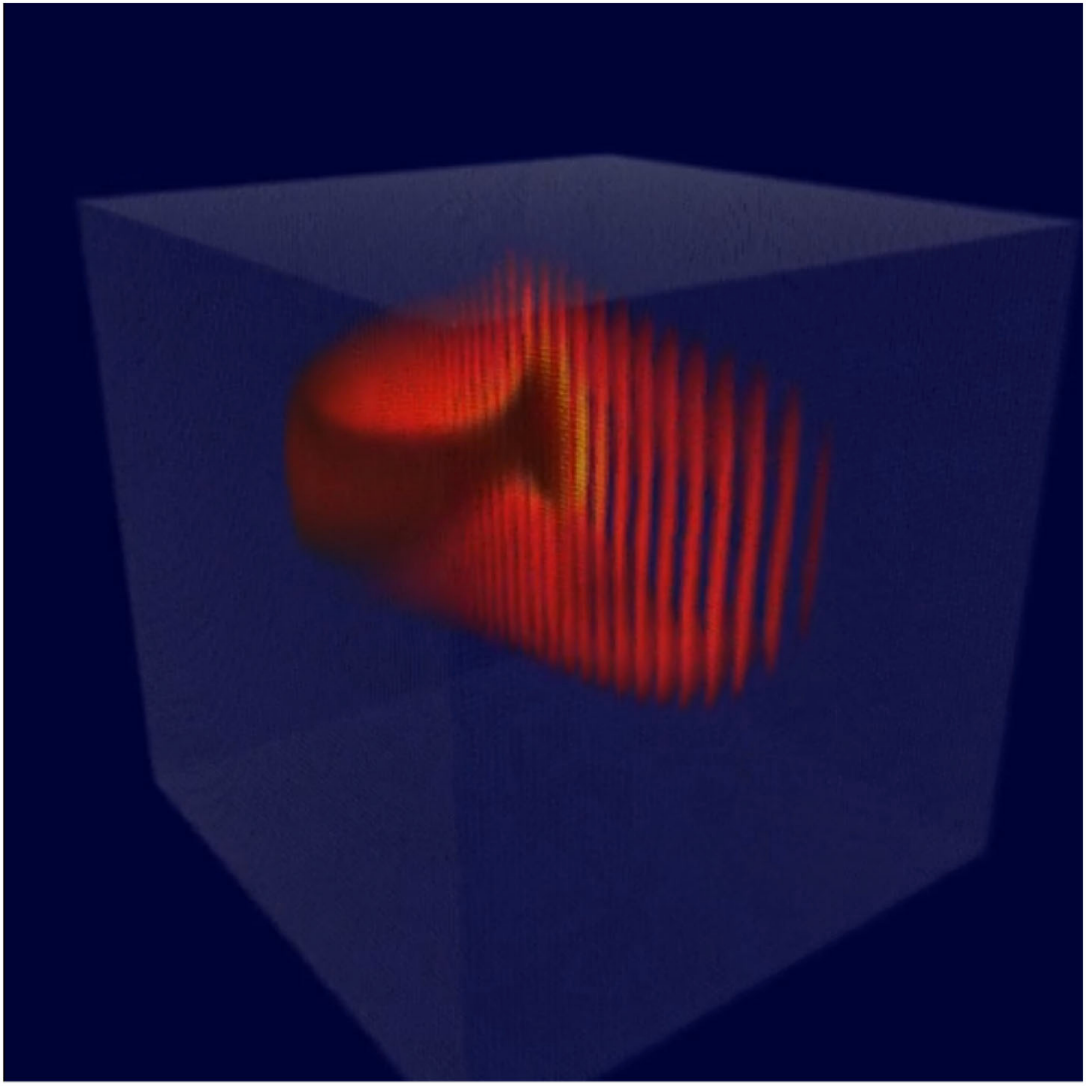
A density plot of a population of Hindmarsh-Rose neurons. The density is contained in a three dimensional volume such that each axis represents one of the time-dependent variables of the model. The volume has been rendered from a rotated and elevated position to more easily visualise the density.

A limitation on the MIIND population density technique is that a maximum of two time-dependent variables can be used to describe the underlying neuron model of each population. In the mesh algorithm, for higher dimensions, mesh building would need to be automated but this is not a trivial problem to solve. The grid algorithm, however, is entirely automated and work has been done to extend MIIND for 3D neuron models. Figure 14 shows the 3D density plot of a population of Hindmarsh-Rose neurons in MIIND. The technique used to generate the 2D transition matrices outlined in section 2 extends to N dimensions so there is theoretically no limit to the dimensionality of the underlying neuron model in the grid algorithm. However, both the grid algorithm and mesh algorithm suffer from “the curse of dimensionality” such that with each additional variable, the number of cells to cover the state space increases to the point where the memory and processing requirements are too high. Luckily, a great number of neuron behaviours can be captured with only two or three time-dependent variables with appropriate approximations.

Large networks can be built up quickly in MIIND. To add a node to a simulation file requires just a single line. Integrating the node into the rest of the network with requisite connections is equally convenient. As mentioned, the Potjans-Diesmann model has been implemented as a single cortical column but this is by no means the limit of the size of network which can be built. It is feasible that a patch of cortex made of perhaps hundreds of cortical columns can be simulated efficiently in MIIND. The benefit of such a network would be to demonstrate how cortical columns interact together under different connectivity regimes and inputs as well as providing the ability to quickly and easily “swap out” the underlying neuron model of each population. Typically, LIF is used but adaptive integrate and fire would be a closer approximation to pyramidal neurons in cortex.

## 10. Conclusion

We have presented the mesh and grid algorithms, MIIND's population density techniques for simulating populations of neurons, and given a full account of the software features available to users. While the mesh algorithm was developed some time ago, the grid algorithm which was added to MIIND recently has precipitated a more accessible, user friendly software package. We hope that the explanations given here along with a lower technical barrier to entry will encourage researchers to make use of the tool.

## Data Availability Statement

The original contributions presented in the study are included in the article/Supplementary Material. The MIIND source code and installation packages are available as a github repository at https://github.com/dekamps/miind. MIIND can be installed for use in Python using “pip install miind” on many Linux, MacOS, and Windows machines with python versions >= 3.6. Documentation is available at https://miind.readthedocs.io/.

## Author Contributions

HO and MK contributed to the text of this article. YL, ML, DS, and LD contributed to the development of the population density technique and MIIND software. All authors contributed to the article and approved the submitted version.

## Conflict of Interest

The authors declare that the research was conducted in the absence of any commercial or financial relationships that could be construed as a potential conflict of interest.
