# Supplementary material for: MIIND : A Model-Agnostic Simulator of Neural Populations
Source: Front Neuroinform. 2021 Jul 6;15:614881. doi: 10.3389/fninf.2021.614881 (PMC8291130; doi:10.3389/fninf.2021.614881)
Supplement: Supplementary file 1 [file Data_Sheet_1.PDF]

# Supplementary Material

## 1 MIIND DIRECTORY STRUCTURE

**Listing 1.** The main top level files in the MIIND repository.

```
MIIND_HOME/
    apps/
    cmake-modules/
    doc/
    examples/
    images/
    libs/
    package/
    Publication/
    python/
    CMakeLists.txt
    setup.py
    vcpkg.json
```

Listing 1 shows the top level directory structure of the MIIND code base. *libs* holds the main library code. *apps* contains the ancillary executables such as MatrixGenerator and Projection as well as various tests and C++ examples. *python* contains the full MIIND Python API including the command line interface. *examples* contains a selection of example simulations which can be run using the *miind.run* module or Python. *package* contains the files necessary to generate a debian package and docker image of MIIND. *CMakeLists.txt* is the highest level cmake file for building MIIND, supported by additional scripts in *cmake-modules*. *setup.py* is the Python script for building and installing the MIIND Python package on all platforms. The Python installation on Windows requires the github submodule vcpkg. Finally, *doc*, *images*, and *Publication* hold files required for building the doxygen documentation.

## 2 MIIND LIBRARY DEPENDENCIES

This section lists some of the third party libraries on which MIIND depends and are widely available on multiple platforms. The Python pip packages are designed to be distributed with all requisite dependencies to eliminate the need for any additional installation. However, for standalone functionality: generating, building and running C++ executables, these libraries must be installed in order for MIIND to function. While most are required, some libraries such as ROOT and CUDA are optional but will limit functionality if unavailable. The full list of dependencies is available in the MIIND documentation.

### Root (Optional)

Root (Brun and Rademakers, 1997) is a library which provides useful data analysis and plotting tools for scientific applications. Because Root is not required for any of the major MIIND functionality, it is an optional dependency and can be disabled when building MIIND using the **ENABLE_ROOT** flag. Disabling ROOT removes the ability to use the <*SimulationIO*> block in the XML file for GeomAlgorithm. Root is disabled for the MIIND Python installation.

### Boost

The basic installation of Boost is a "header only" extension of the C++ standard. MIIND uses Boost to support parallel processing tasks such as the MPI functionality and for solving the Poisson master equation. It is also used for timing and other convenience methods. Because MIIND uses Boost for the MPI processing, the pre-built boost-mpi library must be installed before MIIND will function even if MPI is disabled.

### CUDA (Optional)

In order to use the GPGPU implementation of the MIIND population density technique (MeshAlgorithmGroup and GridAlgorithmGroup), the machine running MIIND must have a CUDA enabled graphics card with the requisite CUDA libraries and drivers installed. Set **ENABLE_CUDA** in the MIIND installation to use the vectorised (Group) algorithms. CUDA is enabled in the Linux and Windows Python packages. CUDA is no longer supported on MacOS.

### MPI (Optional)

MPI can be enabled in the MIIND installation with **ENABLE_MPI**. It must be supported by an installed MPI implementation such as OpenMPI or MPICH. MPI is disabled in all Python packages.

### OpenMP (Optional)

OpenMP is another parallel processing solution which runs specified code blocks in parallel across multiple cores. It is recommended that OpenMP is enabled if possible even if the vectorised algorithms are to be used on the GPU. This is because OpenMP is also used to improve the performance of the geometric transition matrix generator. The **ENABLE_OPENMP** flag in the MIIND installation can be used to enable OpenMP. OpenMP is enabled for the MIIND Python package on all platforms.

## 3 CUDA IMPLEMENTATION

The MIIND CUDA implementation performs similar steps to the CPU version but relies on a different code path which uses the VectorisedNetwork class to contain a simulation. The MIIND Python module, *miindsimv*, instantiates (via the *init* function) VectorisedNetwork which is in the MiindLib library. VectorisedNetwork is designed to run a simulation of vectorised population densities only. For each Node defined in the simulation XML, the main class calls addGridNode, addMeshNode or addRateNode depending on the algorithm type. This is a departure from the CPU version which insantiates the algorithms themselves and passes those as parameters to each node.

The connections between nodes is defined using the functions addGridConnection, addMeshConnection and addMeshCustomConnection which instantiate an internal class for each connection type required for vectorised simulations.

Once all nodes and connections are set up, the initOde2DSystem and setupLoop functions are called which organise the separate probability mass vectors and transition matrices of the individual nodes into contiguous data structures (Fig. S1) ready for parallel processing on the GPU. In initOde2DSystem, the probability mass vectors of each Grid and Mesh node are concatenated into a single vector, _vec_mesh. The reversal and reset mappings for each node and a list of refractive times are also generated. These structures are passed to an instance of CudaOde2DSystemAdapter in the CudaTwoDLib library. The transition matrices for the deterministic dynamics of the grid nodes are concatenated into the _csrs vector which will hold all transition matrices required for the simulation. In the setupLoop function, _csrs is further appended with the transition matrices for solving the master equation for each node (both grid and mesh). The _csrs vector along with the total number of connections, timestep and index lists (mapping the network connections to their associated transition matrices) are passed to an instance of CSRAdapter. The two classes CSRAdapter

and CudaOde2DSystemAdapter in the CudaTwoDLib library are responsible for making calls to the Cuda functions defined in CudaEuler. The function singleStep in VectorizedNetwork, performs a single step of the simulation in a similar fashion to the CPU version of the code. The following algorithm steps are performed.

### 3.1 Update the firing rates and Evolve the Deterministic Dynamics

Each connection has an associated queue of length one or more which allows for transmission delays (implemented in the same way as a refractory period as described in section 3 of the main text). The first task of singleStep is to pop the firing rate from the end of each queue to be passed to the associated target populations. As with the algorithm in TwoDLib::MeshAlgorithm, the deterministic dynamics of each node is progressed forward by one time step. By calling CudaOde2DSystemAdapter::Evolve(), the mapping of each cell in each Mesh Node, defined in TwoDLib::Ode2DSystemGroup, is shifted by one along each strip. This mapping is then passed to the graphics card. CudaOde2DSystemAdapter::RemapReversal() calls a serial cuda function defined in CudaEuler and transfers mass according to the reversal mapping which was loaded onto the card earlier.

In order to evolve the deterministic dynamics of the grid nodes, the Grid Node Transform Matrices in the _csrs vector must be applied once to their respective mass vectors.

### 3.2 Performing the Reset Remapping

In both the Grid and Mesh nodes, cells which lie on the membrane potential threshold are identified and, during each time step, mass in these cells must be moved to cells which lie on the reset potential. However, if the underlying neuron model includes a refractory period, this must also be taken into account when resetting the mass. The function, CudaOde2DSystemAdapter::RedistributeProbability(), performs the full process on the graphics card. Fig. S2 shows the memory structure required to perform the reset functionality on the card. _res_to_mass stores the mass in cells at the threshold in the current time step. _res_sum stores the result of summing _res_to_mass and is extracted to the CPU to produce the current average firing rate. The _refractory_mass block holds the refractory queue for each threshold cell. Each refractory queue has length equal to the number of time steps in the refractory period plus one.

In CudaOde2DSystemAdapter::RedistributeProbability(), first the two blocks, _res_to_mass and _res_sum, are zeroed. Next, each value in the refractory queues of _refractory_mass is shifted to the right towards the end of the queue, leaving an empty space at the beginning. CudaEuler::MapResetToRefractory() moves the mass in each threshold cell to the now empty first position in its respective refractory queue. The threshold cell mass values are also copied to _res_to_mass for summing later. The additional position in each refractory queue is required in the case where the refractory period is not an integer multiple of the time step. In this case, only a proportion of the mass at the end of the queue is passed to the required reset cell. The remaining mass is then shifted to the additional cell and passed to the reset cell during the next iteration. Therefore, in each iteration, a reset cell receives a proportion of mass from the end cell of the queue and $1 - proportion$ of the mass from the additional cell. Finally, sumReset is called and the result of summing _res_to_mass is passed to _res_sum.

### 3.3 Solve the Master Equation for the Stochastic Noise Process

For all nodes, using both the grid and mesh algorithms, solving the spread of mass across the state space due to incoming Poisson distributed spikes is achieved through multiple applications of the associated transition matrices. The mass vector for each of the nodes in _vec_mesh are multiplied by each of the transition matrices in _csrs corresponding to incoming connections.

The VectorizedNetwork variable _master_steps, which can be set by the user in the XML file, defines how many times the matrix multiplication is performed. _master_steps must be large enough to keep the Euler numerical integration stable. The stability is dependent on the input rate of the connection and the size of the transition for each cell (which in turn is dependent on the efficacy and local dynamics of the mesh).

For this part of the process, the grid and mesh algorithms only differ in the way that the transition matrices are generated. As discussed, the mesh algorithm *.mat* files are pre-generated and passed as a static data structure. For the grid algorithm, the transitions matrix can be generated at simulation time due to the regularity of the grid cells which allows for more flexibility in their definition.

# 4 GRID ALGORITHM SPECIALISATIONS

The regularity of the grid cells in the grid algorithm allows MIIND to calculate the transition matrix for solving the Poisson master equation at run time. When using GridAlgorithms, then, the connections can be somewhat dynamic, for example, depending on the average membrane potential of the In or Out populations. Because the way that the transition matrix is calculated can be very model specific, this must be defined in a C++ extension to the existing code base. However, two model cases are available for use "as is" or as a template. Example simulations of both of these models is available in the model archive in the code repository.

## 4.1 Minimal Motor Neuron

The minimal motor neuron model by Booth and Rinzel Booth and Rinzel (1995) is a two compartment model with a soma and dendrite part. Each part is comprised of a two dimensional dynamical system including a term which integrates the membrane potential from the other compartment.

An approximation of a population of these motor neurons can be simulated in MIIND by using the specialised algorithm GridSomaDendriteAlgorithm to simulate the behaviour of a population of somas and a population of dendrites. This is clearly different to a population of individually coupled pairs which is why it is an approximation. To account for the term in each of the compartments representing the membrane potential of the opposing part, MIIND uses the average membrane potential of each population. Two connections are defined between the GridSomaDendriteAlgorithm populations to pass the shared average membrane potential values. The connections are identified with a type attribute equal to "SomaDendrite" and a "conductance" which represents the degree of coupling of the two populations as described by Booth and Rinzel (1995). A specialised solver for the Poisson master equation solver, MasterGridSomaDendrite takes the conductance value of the connection and multiplies this by the difference between the membrane potential of each cell and the average membrane potential of the other population (provided via the connection). The GridSomaDendriteAlgorithm is currently not available for use with the GPGPU.

## 4.2 2D Epileptor

The 2D Epileptor neuron model (Proix et al., 2014, 2017) is a reduced version of the full Epileptor model (Jirsa et al., 2014) developed for use with The Virtual Brain for investigating the propagation of epileptic seizures across the whole brain. As with the GridSomaDendriteAlgorithm, the model calls for populations connected using more than just the average firing rate or membrane potential. The efficacies of two Epileptor populations are calculated using the K and tau parameters which must be provided as attributes to connections with type "epileptor". How these values are used is described by Proix et al. (2014). Unlike the GridSomaDendriteAlgorithm, a separate algorithm type is not defined for the Epileptor as the integration of the population is no different to any other grid. The difference is only required at the level of the connections which must be set with the correct attributes.

## 5 GENERATING MARGINAL DENSITY PLOTS

The density files generated by MeshAlgorithm and GridAlgorithm (and variants) can be used to plot both the joint distribution of the two time dependent variables and the marginal densities showing the distribution across each individual dimension. The marginal density is essentially a histogram of the probability mass along each axis of the state space. The required dimension is split into equal-sized bins and, at a given simulation time, the total mass inside each bin is summed. For densities from MeshAlgorithm, mesh cells may span multiple bins so the correct proportion of probability mass in each cell must be attributed to the correct bin. This is also true of the GridAlgorithm although the cells are more regular. In order to calculate the attribution of mass to bins, yet again, the geometric method for generating transition matrices can be used. The area of overlap of a cell with each bin is calculated as described in section 2.1 of the main text and used to define the proportion to be attributed to that bin. The *Projection* program which is a separate executable similar to *GenerateMatrix* implements the algorithm for calculating the transition matrix which is then stored in a *.projection* file in the working directory of the simulation XML file. The projection file is then used for calculating the specific marginal density for a specific time in the simulation. When the **plot-marginals** command is called in *miind.miindio*, if the projection file does not exist or the mesh has been modified since the projection file was last generated, the *Projection* executable is called first then the specific marginal density can be calculated for the requested time point. As with plotting the full density, the time point parameter passed to **plot-marginals** must match a density file in the output directory of the simulation which therefore requires that the simulation includes a *<Density>* entry in the *<Recording>* section of the XML for the given population node. In order to plot the marginals of a given node in the simulation, a projection file for the associated model file must have been generated.

## 6 BUILDING A MESH FOR MESH ALGORITHM

This section gives more detail on how to build a mesh for use with the mesh algorithm in MIIND. Like the grid algorithm, theoretically any model can be build in this way. However, there are a number of subtleties to building a mesh which can require a deep understanding of the required model as well as an understanding of numerical integration techniques.

To define a single strip in the mesh, the mesh developer picks two nearby points in state space, usually some distance from the volume of space where the majority of probability mass will end up during simulation. Using a finite difference solver, stepping forward from both points according to the neuron model definition until a predetermined fixed time step is reached generates two new points. Together with the starting points, these form the first quadrilateral cell of the strip. By continuing to step forward with the solver, more cells are added to the strip. The developer should use their knowledge of the neuron model to pick starting points which lead to the strip extending into the state space where the majority of mass will stay during simulation. If the model contains a stationary point, some strips will likely approach it. As they do so, the cells will get smaller. At an appropriate point, the developer should terminate the generation of each strip such that the space between the end of the strip and the stationary point is minimised while avoiding too many small cells. The smaller and more numerous the cells get, the slower the simulation will be, so this is a balance between accuracy and performance. The potential difficulties and strategies to mitigate them are discussed in great detail by De Kamps et al. (2019). By repeating this process for many starting points around the state space, many strips can be generated which cover the space and form the whole mesh. In some instances, if the dynamics approach some discontinuity or go to infinity, the solver will fail to generate more points for a strip. Due to the difference between one edge of the strip and the other, this might cause the cells to become extremely shear. In this scenario, it might be more appropriate to backwards-integrate from starting points in this region. Further advice for mesh building is listed in section 7. The need to sometimes

cleverly pick the starting points of strips makes this process difficult to fully automate. Even changes to parameters of the same neuron model can yield very different dynamics requiring a different mesh. Once all of the vertices of the mesh have been generated they must be stored in a *.mesh* file with the format in listing 2. The order in which the points are defined from left to right for each strip defines the direction that mass moves along the strip during the simulation. All strips must be generated with the same time step which will be used in the simulation. For convenience, the two dimensions are often labelled v for the horizontal axis and h for the vertical axis although they could represent any variable.

**Listing 2.** The format for mesh files.

```
#### first line is ignored
[timestep used to generate the strips]
[tab delimited v coordinates of points along the lower side of strip 0]
[tab delimited h coordinates of points along the lower side of strip 0]
[tab delimited v coordinates of points along the upper side of strip 0]
[tab delimited h coordinates of points along the upper side of strip 0]
closed
[tab delimited v coordinates of points along the lower side of strip 1]
[tab delimited h coordinates of points along the lower side of strip 1]
[tab delimited v coordinates of points along the upper side of strip 1]
[tab delimited h coordinates of points along the upper side of strip 1]
closed
...
[tab delimited v coordinates of points along the lower side of strip n]
[tab delimited h coordinates of points along the lower side of strip n]
[tab delimited v coordinates of points along the upper side of strip n]
[tab delimited h coordinates of points along the upper side of strip n]
closed
end
```

Two further files are required before MIIND can automatically perform the remaining preprocessing steps to begin simulating a population. The mesh developer should generate a *.stat* file which defines additional quadrilaterals which will contain probability mass in the simulation which does not move along any strip. This is useful for approximating mass which has settled at a stationary point in the state space. Often there is only one stationary cell and this is defined as the starting cell for the simulation, where all probability mass initially resides. The format for the stat file is demonstrated in listing 3 which shows the definition of a single stationary square at location[0.0,0.0] with width = 1e-05 and height = 5.0. Each quadrilateral contains the v values then the h values for all four points. As many stationary cells as are required can be added with further *<Quadrilateral>* elements. The initial mass will always be in cell [0,0].

**Listing 3.** The format for .stat files.

```
<Stationary>
<Quadrilateral><vline>0.0 0.0 1e-05 1e-05</vline><wline>0 5.0 5.0 0</wline></Quadrilateral>
</Stationary>
```

The default behaviour for mass which reaches the end of a strip is to loop back to the initial cell. Sometimes this never happens if the strip passes through a threshold. However, for strips which approach a stationary point, the desired behaviour should be for mass to leave the end of the strip and transition to the stationary cell which surrounds the stationary point. The developer must define this behaviour in the *.rev* file. This file is similar to the transition matrix files we will see later and lists coordinates of a source cell, a target cell, and then a proportion value. Intuitively, a transition from the last cell in a strip to a stationary

cell should reference these two cells. However, in the MIIND simulation loop, mass is shifted down each strip before the reversal mapping is applied. This means that the mass to be transferred to the stationary cell will reside in the initial cell of the strip as illustrated in Fig. S3. The mapping must therefore define a transition from that first cell to the stationary cell with proportion = 1.0.

Listing 4 shows the format for the *.rev* file. There are other uses for the reversal file, such as when two strips are used to describe consecutive parts of a single trajectory such that when mass reaches the end of one strip it must be passed to the starting cell of the next. Another case is when one strip defines a limit cycle and approaching strips must be "stitched" so that mass can transition correctly. These scenarios are discussed in section 7.

**Listing 4.** An example reversal file which describes two transitions from the first cell in strip 1 and the first cell in strip 2 to a stationary cell at 0,0 with proportion 1.0.

```
<Mapping type="Reversal">
1,0   0,0   1.0
2,0   0,0   1.0
</Mapping>
```

The resulting three files from this process can be used by the **generate-model** command in the MIIND CLI to build a *.model* file.

### 6.1 Using the Monte Carlo Technique to Generate Transition Matrices

The main article describes how a Monte Carlo method can be used to generate transition matrices for the mesh algorithm. Points are placed in each cell and translated according to the required efficacy or jump. A proportionate transition value is given for each cell which contains one or more translated points. However, the method generates points which can be translated outside of the mesh, where there are no cells. This will happen for many cells on the edge of the mesh and for cells which are near any gaps in the mesh (for example, near a separatrix). These points must be accounted for so that the transition proportions of each cell sum to one. If a cell's transition does not sum to one, every time it is applied during the simulation, the probability mass in that cell will be removed from the system. In the event that a point falls outside the mesh, it is assigned to the cell with the nearest Euclidean distance. In order to speed up this search, the user must provide "fiducial regions" to narrow down the number of cells to test. To store these regions, an additional *.fid* file is required which can be created using the **generate-empty-fid** command in *miind.miindio*. It is initially empty but will need to be populated once the transition matrix has been generated for the first time. Although finding the closest cell is an approximation being made about the movement of mass, in many cases the gap in the mesh is small or lies along a separatrix in the state space such that a neuron in the gap would translate to its nearest cell very quickly anyway. Furthermore, the mesh itself should be designed to cover enough of the state space such that, during the simulation, large amounts of mass do not enter those cells which have transitions which extend beyond the edges. That is, the majority of mass should remain away from the edges of the mesh during simulation so any error introduced by this process affects only a very small proportion of the PDF. When **generate-matrix** is called, the lost points are recorded in the *.lost* file. If the lost file is empty, this means that there will be no lost mass during the simulation and the *.mat* file is ready to use. If the file does contain points, the user must populate the fiducial file with regions in which MIIND can search for appropriate cells to assign to each point. The **generate-matrix** command must then be run once again. *miind.miindio* provides a command, **lost**, for identifying missing points and drawing these fiducial regions. Calling **lost** with the full name of the *.lost* file will open a graphical user interface window where the user can repeatedly click the location of four corners to generate fiducial regions. The regions should be positioned so that they cover all areas of

the state space where there are lost points. The edges of the regions should be far enough away from the lost points such that a new set of random points will not fall outside the edges. If the edge of a region is very close to a point, it is possible that the next time **generate-matrix** is run, that point could fall slightly outside the region and be lost again. However, the edges of the region must be close enough to the points to limit the number of cells whose distance must be checked. Fig. S4 shows an example of fiducial regions drawn in the **lost** interface window. Once all points have been surrounded by fiducial regions, the user can double click or press enter to close the interface window and write the regions to the *.fid* file. Re-running **generate-matrix** should now yield an empty *.lost* file, guaranteeing that probability mass is conserved throughout the simulation.

# 7 MESH BUILDING TIPS

The following are a set of methods to aid in the building of meshes for use with MeshAlgorithm (see Fig. S5 for illustrations).

## 7.1 Backwards Integrating

Many models have a volume of state space where trajectories quickly approach infinity with the expectation that the neuron will be reset above a certain threshold. In these models strips can become extremely shear as one side of the strip increases quicker than the other (Fig. S5A). All points will also quickly become too high to be represented and the integration of the trajectories will fail. Sometimes it is possible to simply cut short all strips before they reach a certain threshold where these problems occur. However, an alternative is to choose the starting locations of the trajectories at some high value beyond the threshold and backwards integrate as shown in Fig. S5B.

## 7.2 Using Nullclines

The nullclines of a model can give a good indication of where starting positions should be placed (Fig. S5C). Where trajectories flow away from a nullcline, starting positions can be chosen just slightly on either side such that strips will travel away. The gap that is left along the nullcline itself is unimportant as the instability on that line would mean that neurons will quickly move to one side or the other. Likewise, where trajectories approach a nullcline, it can be useful to choose starting points on either side and backwards integrate.

## 7.3 Strip Stitching

Strip stitching involves using the reversal mapping to pass mass from the end of one strip to the start of another. this can be useful in a number of situations. For example, if the model to be built has a limit cycle (such as the FitzHugh-Nagumo neuron model), it is often preferable to have a single strip which follows the cycle and have strips which approach the limit cycle but stop short of the cycle strip itself. The reversal mapping can then be used to shift mass form the end each strip to the nearest cell on the cycle strip. This prevents extreme shearing of the cells as they begin to wind around the limit cycle. Often there are areas of state space where cells are generally large and regular. Even if these are situated among areas of high cell density (for example, within the bend of a nullcline), it can be beneficial to pick a line or curve of starting points which passes through the area of low density then both forward and backwards integrate from each point. Strip stitching must be employed to attach the two opposing strips together for each of the starting points as shown in Fig. S5D. As with reversal mappings for transferring mass to a stationary cell, the mapping must be defined between the first cell of strip one and the first cell of strip two to account for the shift of mass occurring before the mapping is performed during each time step.

# 8 PARAMETER SWEEPS USING VARIABLES

As discussed in section 4.6 of the main text, the use of Variables in the XML file supports convenient methods for performing parameter sweeps. As an example, suppose that an experiment is carried out to observe the effect of noise on a population of LIF neurons. As described in section 9 of the main text, two quantities can be set to control the mean and variance of the change to the probability density: the input rate and the efficacy. To perform a parameter sweep across these two quantities, the appropriate variables can be declared and added to the XML as in listing 5.

**Listing 5.** An abridged version of an XML file with two variables to control the input rate and efficacy of a population of LIF neurons.

```
...
<Variable Name="RATE">1500.0</Variable>
<Variable Name="EFFICACY">0.01</Variable>
...
<Algorithm type="MeshAlgorithm" name="E" modelfile="lif.model" >
<TimeStep>0.001</TimeStep>
<MatrixFile>lif_0.01_0_0_0_.mat</MatrixFile>
<MatrixFile>lif_0.02_0_0_0_.mat</MatrixFile>
...
<MatrixFile>lif_0.1_0_0_0_.mat</MatrixFile>
</Algorithm>
<Algorithm type="RateFunctor" name="ExcInput">
<expression>RATE</expression>
</Algorithm>
<Nodes>
<Node algorithm="E" name="LIF" type="EXCITATORY_DIRECT" />
<Node algorithm="ExcInput" name="Inp" type="NEUTRAL" />
</Nodes>
<Connections>
<Connection In="Inp" Out="LIF">1 EFFICACY 0</Connection>
</Connections>
<Reporting>
...
```

When calling *miind.run* with only the XML file as a parameter, the simulation will use the default values for RATE and EFFICACY in the Variable definitions. Calling *miind.run* with additional parameters setting a name for the XML file and key-value pairs for the two variables, will override the default values.

```
$ python -m miind.run lif.xml RATE=750.0 EFFICACY=0.02
```

## 8.1 Using the MIIND Python module to Perform Sweeps

An alternative to multiple *miind.run* calls is to generate a single Python script which calls *init* with the requisite variable definitions as parameters. The XML file in listing 5 can be altered so that the rate is controlled by a Python external connection, and the efficacy remains a variable to be set in the Python script.

**Listing 6.** An abridged version of an XML file to be used in a Python script with a single variable to control the efficacy.

```
...
<Variable Name="EFFICACY">0.01</Variable>
...
<Algorithm type="MeshAlgorithm" name="E" modelfile="lif.model" >
```

```
<TimeStep>0.001</TimeStep>
<MatrixFile>lif_0.01_0_0_0_.mat</MatrixFile>
<MatrixFile>lif_0.02_0_0_0_.mat</MatrixFile>
...
<MatrixFile>lif_0.1_0_0_0_.mat</MatrixFile>
</Algorithm>
<Nodes>
<Node algorithm="E" name="LIF" type="EXCITATORY_DIRECT" />
</Nodes>
<Connections>
<IncomingConnection Node="LIF">1 EFFICACY 0</IncomingConnection>
<OutgoingConnection Node="LIF"/>
</Connections>
<Reporting>
...
```

*init* can now be called in the Python script with the additional parameter to set the efficacy. Variable values must be passed as strings.

```
miind.init(number_of_nodes, sim_filename, EFFICACY=str(efficacy))
```

A Python program which takes the efficacy and input rate as parameters can be called multiple times and in parallel for high performance parameter sweeps. Alternatively, the simulation can be run multiple times in the same script in a loop with different parameters. The example parameter sweep scenario shown here is available in the *examples/lif_python_sweep* directory.

# 9 CODE LISTINGS

## 9.1 Izhikevich Mesh and Grid Generators

The following MATLAB script, *izh.m*, can be used to generate a mesh file for use with a MeshAlgorithm population in MIIND. The mesh describes the dynamics of the Izhikevich Simple Model (Izhikevich, 2003) in its bursting configuration. The supporting *izhikevich.m* script defines the neuron model itself.

**Listing 7.** File izh.m for generating an Izhikevich Simple Model mesh.

```
%#ok<*NOPTS>
% Reset should be -50, threshold is -30,  d = 2

revId = fopen('izh.rev', 'w');
fprintf(revId, '<Mapping Type="Reversal">\n');

outId = fopen('izh.mesh', 'w');
fprintf(outId, 'ignore\n');
fprintf(outId, '1\n');

formatSpec = '%.12f ';
strip = 0;
for u0 = -10.5:1:15.5
    tspan = 0:0.1:250;
    v0 = -150;

    [t1,s1] = ode45(@izhikevich, tspan, [v0 u0]);
    [t2,s2] = ode45(@izhikevich, tspan, [v0 u0+1]);
```

```
    x1 = s1(:,1);
    y1 = s1(:,2);

    x2 = s2(:,1);
    y2 = s2(:,2);

    svs = [];
    sus = [];
    for t = 1:min(length(x2),length(x1))
        a = x1(t);
        b = y1(t);
        c = x2(t);
        d = y2(t);

        svs = [svs, x1(t)];
        sus = [sus, y1(t)];

        plot([a c],[b d],'k');
        hold on;
    end

    fprintf(outId, formatSpec, svs);
    fprintf(outId, '\n');
    fprintf(outId, formatSpec, sus);
    fprintf(outId, '\n');

    fprintf(revId, '%i,%i\t%i,%i\t%f\n', strip, length(svs)-1, 0,0, 1.0);
    strip = strip + 1;

    axis equal
    xlim([-150 10]);
    ylim([-15 15]);

    title('Izhikevich');
    ylabel('u');
    xlabel('v');

    plot(x1,y1,'k');
    hold on;
end

fprintf(outId, 'closed\n');
%-55.5 -53.7
for v0 = -53.7:0.4:-40
    tspan = 0:0.1:200;

    eps = 0.4;

    I = 15;
    u0 = 0.04*v0^2 + 5*v0 + 140 + I + eps;

    u0_next = 0.04*(v0+0.4)^2 + 5*(v0+0.4) + 140 + I + eps;

    [t1,s1] = ode45(@izhikevich, tspan, [v0 u0]);
```

```
    [t2,s2] = ode45(@izhikevich, tspan, [v0+0.4 u0_next]);

    x1 = s1(:,1);
    y1 = s1(:,2);

    x2 = s2(:,1);
    y2 = s2(:,2);

    svs = [];
    sus = [];
    for t = 1:min(length(x2),length(x1))
        a = x1(t);
        b = y1(t);
        c = x2(t);
        d = y2(t);

        svs = [svs, x1(t)];
        sus = [sus, y1(t)];

        plot([a c],[b d],'k');
        hold on;
    end

    fprintf(outId, formatSpec, svs);
    fprintf(outId, '\n');
    fprintf(outId, formatSpec, sus);
    fprintf(outId, '\n');

    fprintf(revId, '%i,%i\t%i,%i\t%f\n', strip, length(svs)-1, 0,0, 1.0);
    strip = strip + 1;

    plot(x1,y1,'k');
    hold on;

    axis equal
    xlim([-150 10]);
    ylim([-15 15]);

    title('Izshikevich');
    ylabel('u');
    xlabel('v');
end

fprintf(outId, 'closed\n');
%-56.1  -62.0
for v0 = -62.0:1:-40
    tspan = 0:0.1:120;

    eps = 0.2;

    I = 15;
    u0 = 0.04*v0^2 + 5*v0 + 140 + I + eps;

    u0_next = 0.04*(v0+1)^2 + 5*(v0+1) + 140 + I + eps;
```

```
    [t1,s1] = ode45(@izhikevich, tspan, [v0 u0]);
    [t2,s2] = ode45(@izhikevich, tspan, [v0+1 u0_next]);

    x1 = s1(:,1);
    y1 = s1(:,2);

    x2 = s2(:,1);
    y2 = s2(:,2);

    svs = [];
    sus = [];
    for t = 1:min(length(x2),length(x1))
        a = x1(t);
        b = y1(t);
        c = x2(t);
        d = y2(t);

        svs = [svs, x1(t)];
        sus = [sus, y1(t)];

        plot([a c],[b d],'k');
        hold on;
    end

    fprintf(outId, formatSpec, svs);
    fprintf(outId, '\n');
    fprintf(outId, formatSpec, sus);
    fprintf(outId, '\n');

    fprintf(revId, '%i,%i\t%i,%i\t%f\n', strip, length(svs)-1, 0,0, 1.0);
    strip = strip + 1;

    plot(x1,y1,'k');
    hold on;

    axis equal
    xlim([-150 10]);
    ylim([-15 15]);

    title('Izhikevich');
    ylabel('u');
    xlabel('v');
end

fprintf(outId, 'closed\n');

fprintf(outId, 'end\n');

fclose(outId);

fprintf(revId, '</Mapping>\n');
fclose(revId);
```

```
outId = fopen('izh.stat', 'w');
fprintf(outId, '<Stationary>\n');
fprintf(outId, '<Quadrilateral><vline>-80.05 -80.05 -79.95 -79.95</vline><wline>4.95 5.05 5.05 4.95</
    ↪ wline></Quadrilateral>\n');
fprintf(outId, '</Stationary>\n');
fclose(outId);
```

**Listing 8.** File izhikevich.m.

```
function izh = izhikevich(t, ws)
    a = 0.02;
    b = 0.2;
    I = 15;

    v = ws(1);
    u = ws(2);

    v_prime = ((0.04*v^2) + (5*v) + 140) - u + I;
    u_prime = a * ((b * v) - u);

    izsh = [v_prime; u_prime];
end
```

## 9.2 Generating the Izhikevich grid for GridAlgorithm

```
import grid_generate

def izh(y,t):
    v = y[0];
    w = y[1];

    v_prime = 0.04*v**2 + 5*v + 140 - w + 10
    w_prime = 0.02 * (0.2*v - w)

    return [v_prime, w_prime]


grid_generate.generate(izh, 0.1, 0.001, 1e-4, 'izh', -30.0, -50.0, 2.0, -85.0, -10.0, -15.0, 20.0, 500,
    ↪ 500)
```

## 9.3 Command Line Interface Command Listing

The following listing can be found by typing the command, **help**, into the command line interface provided by *miind.miindio*.

```
help                      : Get this help menu.
quit                      : Close the UI.

***** Commands for Creating and Running Simulations *****

sim      : Set the current simulation from an xml file or generate a new xml file.
models   : List all model files used by the current simulation.
settings : Set certain persistent parameters to match your MIIND installation (ENABLE ROOT, CUDA).
submit   : Generate and build (make) the code from the current simulation.
run      : Run the current submitted simulation.
submit-python  : Generate and build (make) a shared library for use with python from the current
    ↪ simulation.
```

```
***** Commands for Analysing and Presenting Completed Simulations *****

rate   : Plot the mean firing rate of a given node in the current simulation.
avgv   : Plot the mean membrane potential of a given node in the current simulation
plot-density    : Plot the 2D density of a given node at a given time in the simulation.
plot-marginals  : Plot the marginal densities of a given node at a given time in the simulation.
generate-density-movie  : Generate a movie of the 2D density for a given node in the simulation.
generate-marginal-movie : Generate a movie of the Marginal densities for a given node in the simulation.

***** Commands for Building New Models and Matrix Files *****

generate-model     : Generate a model file from existing mesh, rev and stat files.
generate-empty-fid : Generate a stub .fid file.
generate-matrix    : Generate a matrix file from existing model and fid files.
regenerate-reset   : Regenerate the reset mapping for an existing model.
lost               : Open the fiducial tool for capturing lost points.
generate-lif-mesh  : Helper command to build a LIF neuron mesh.
generate-qif-mesh  : Helper command to build a QIF neuron mesh.
generate-eif-mesh  : Helper command to build a EIF neuron mesh.
draw-mesh          : Draw the mesh described in an existing .mesh file.
```

## 9.4 Figures

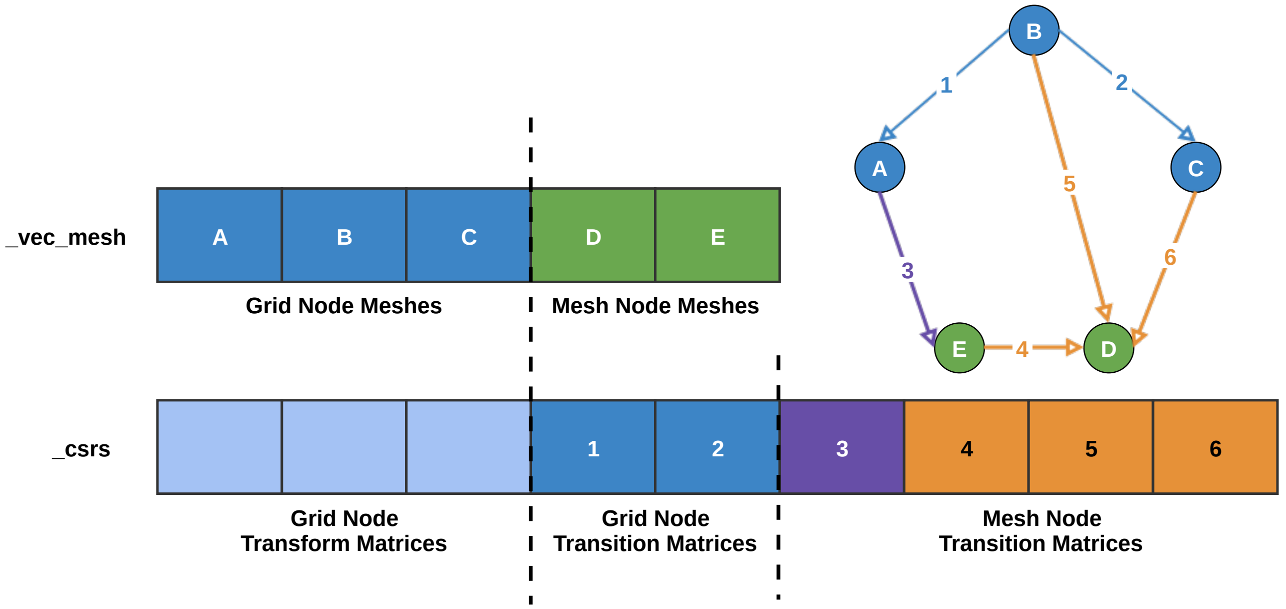


**Figure S1.** The data structures used for the vectorised form of MeshAlgorithm and GridAlgorithm. Nodes A, B, and C are GridAlgorithm nodes. Nodes D and E are MeshAlgorithm nodes. _vec_mesh holds the discretised probability density functions for the grid nodes and the mesh nodes. _csrs holds the transition matrices which produce the deterministic dynamics of the grids and the matrices required to solve the Poisson master equation for each connection in the network.

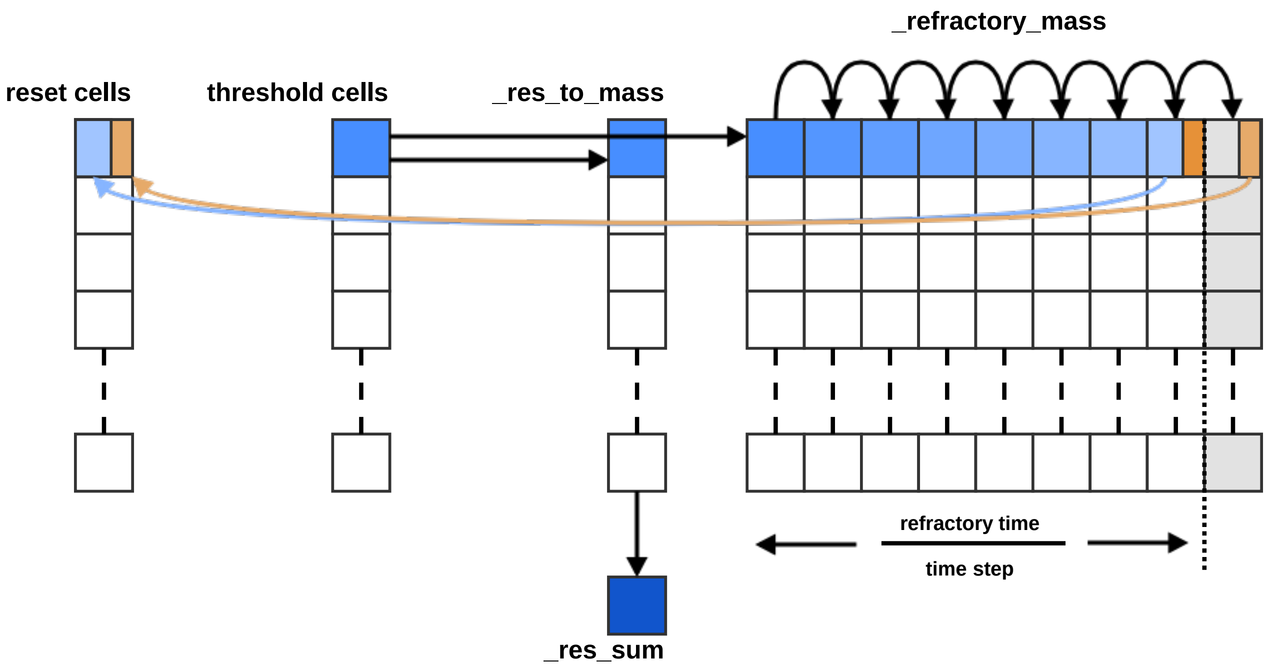


**Figure S2.** The vectorised implementation of reset functionality is very similar to that of the CPU version. However, there is an additional data structure, _res_to_mass, which initially holds a copy of the mass in the threshold cells after each iteration. The structure is used to sum the cells in a parallel manner and the result is stored in _res_sum which can then be extracted from the GPU and used to calculate the average firing rate for each population.

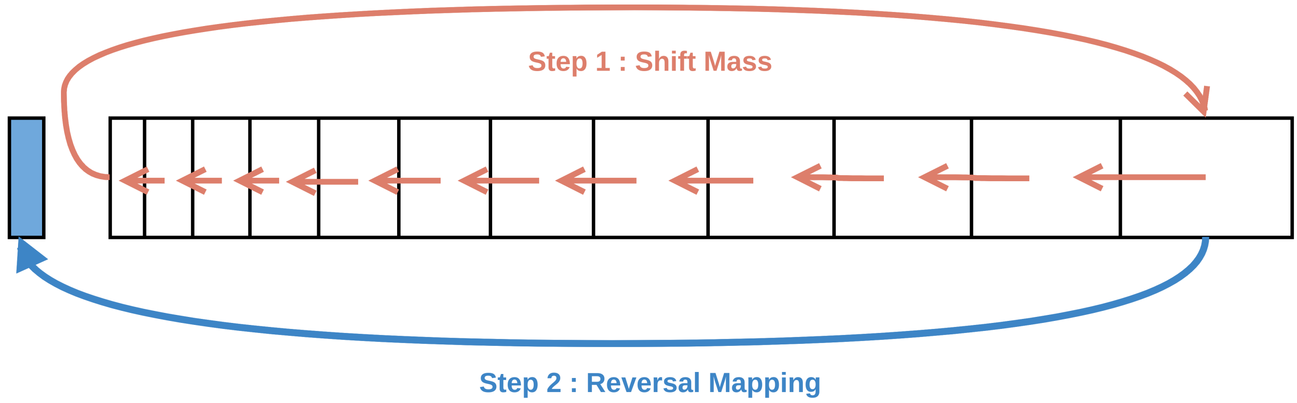


**Figure S3.** Reversal mapping for a strip which approaches a stationary cell. The aim is to take probability mass from the end of the strip to the stationary cell. During MIIND's simulation loop, probability mass is first shifted one cell down the strip. The mass in the last cell is shifted to the first cell. The second step is for the reversal mapping to be applied. Therefore, the mapping should take mass from the first cell of the strip to the stationary cell.

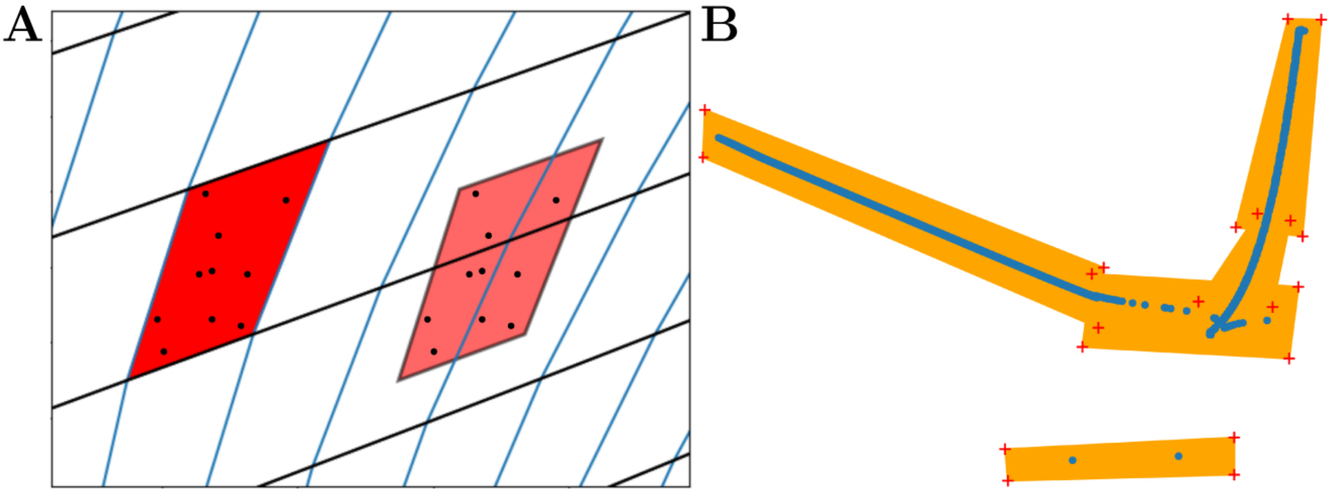


**Figure S4.** (A) To calculate the transitions of each cell due to a single incoming spike, the proportion of neurons that will end up in each target cell must be calculated. The Monte Carlo method of calculating the proportions is to take a number of points randomly placed in the source cell and translate them by the efficacy amount. The geometric solution is to translate the source cell vertices themselves and calculate the area overlap with target cells. (B) When using the Monte Carlo method, cells may be translated outside of the mesh but must be accounted for. The "lost" tool displays the missing points after **generate-matrix** has been called once. The user may then draw quadrilaterals (fiducial areas) around where the points lie. During the second call to **generate-matrix**, these areas will be used to search for nearby mesh cells to which the missing points can be attributed.

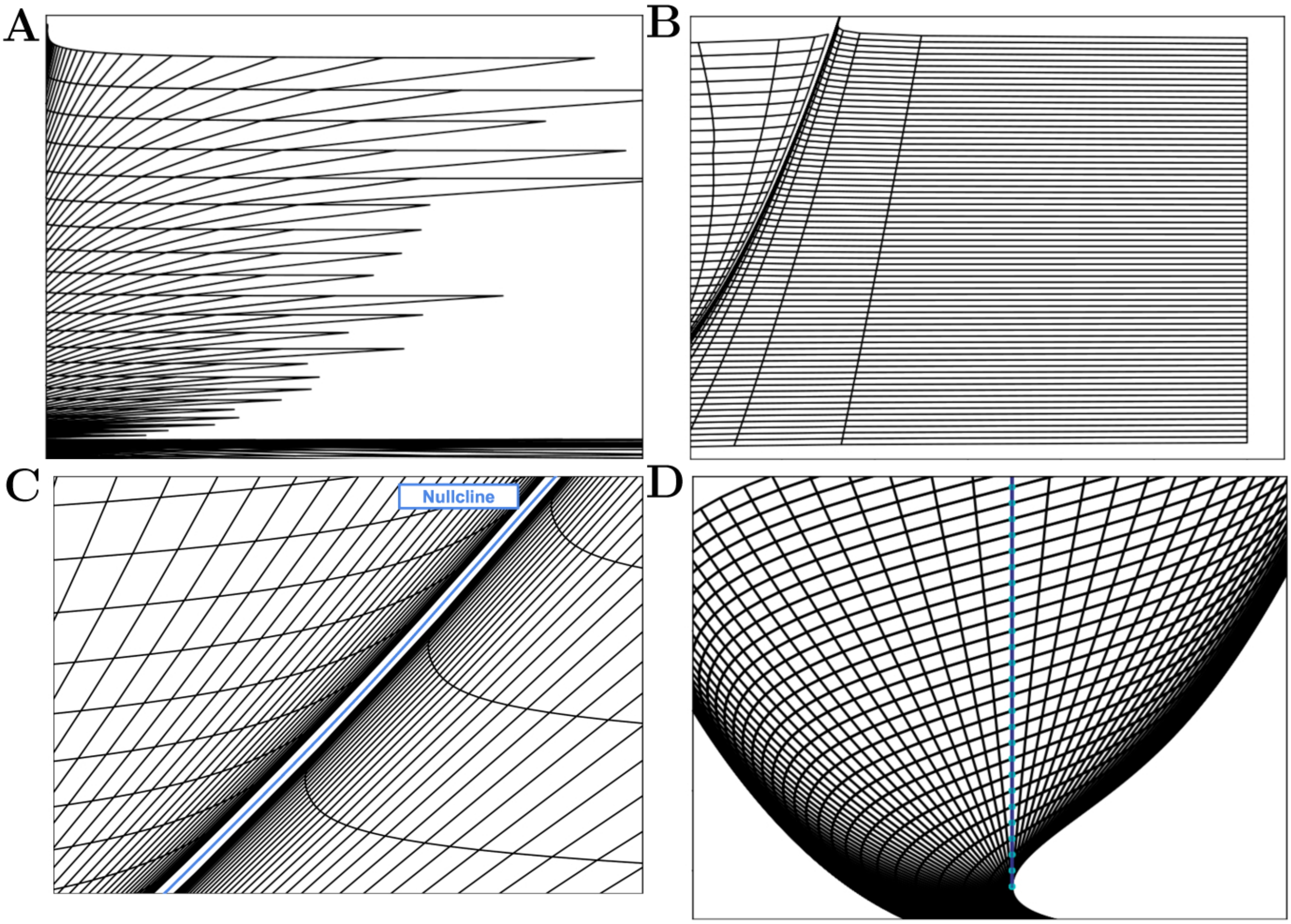


**Figure S5.** (A) As trajectories moving off to the right approach infinity, each strip is cut off at a different place producing a “feathering” effect which can make it difficult to identify threshold cells. (B) By picking starting points at a high value then backwards integrating, the feathering is eliminated. (C) Picking starting points for strip trajectories on either side of a nullcline and integrating away produces only a small gap where the nullcline itself lies. (D) An example of an area of low cell density split into two by a line of starting points (in blue). This technique handles the transition from high to low density cells on either side.

## REFERENCES


Booth, V. and Rinzel, J. (1995). A minimal, compartmental model for a dendritic origin of bistability of motoneuron firing patterns. *Journal of computational neuroscience* 2, 299–312

Brun, R. and Rademakers, F. (1997). Root—an object oriented data analysis framework. *Nuclear Instruments and Methods in Physics Research Section A: Accelerators, Spectrometers, Detectors and Associated Equipment* 389, 81–86

De Kamps, M., Lepperød, M., and Lai, Y. M. (2019). Computational geometry for modeling neural populations: From visualization to simulation. *PLoS computational biology* 15, e1006729

Izhikevich, E. M. (2003). Simple model of spiking neurons. *IEEE Transactions on neural networks* 14, 1569–1572

Jirsa, V. K., Stacey, W. C., Quilichini, P. P., Ivanov, A. I., and Bernard, C. (2014). On the nature of seizure dynamics. *Brain* 137, 2210–2230

Proix, T., Bartolomei, F., Chauvel, P., Bernard, C., and Jirsa, V. K. (2014). Permittivity coupling across brain regions determines seizure recruitment in partial epilepsy. *Journal of Neuroscience* 34, 15009–15021

Proix, T., Bartolomei, F., Guye, M., and Jirsa, V. K. (2017). Individual brain structure and modelling predict seizure propagation. *Brain* 140, 641–654
